# Sceptic: pseudotime analysis for time-series single-cell sequencing and imaging data

**DOI:** 10.1186/s13059-025-03679-3

**Published:** 2025-07-17

**Authors:** Gang Li, Hyeon-Jin Kim, Sriram Pendyala, Ran Zhang, Jean-Philippe Vert, Christine M. Disteche, Xinxian Deng, Douglas M. Fowler, William Stafford Noble

**Affiliations:** 1 Department of Genome Sciences, University of Washington, Seattle, 98115 USA; 2 eScience Institute, University of Washington, Seattle, 98115 USA; 3 Medical Scientist Training Program, University of Washington, Seattle, USA; 4 Owkin, Paris, 75010 France; 5 Department of Laboratory Medicine and Pathology, University of Washington, Seattle, 98115 USA; 6 Department of Medicine, University of Washington, Seattle, 98115 USA; 7 Department of Bioengineering, University of Washington, Seattle, 98115 USA; 8 Paul G. Allen School of Computer Science and Engineering, University of Washington, Seattle, 98115 USA

## Abstract

Several computational methods have been developed to construct single-cell pseudotime embeddings for extracting the temporal order of transcriptional cell states from time-series scRNA-seq datasets. However, existing methods suffer from low predictive accuracy, and this problem becomes even worse when we try to generalize to other data types such as scATAC-seq or microscopy images. To address this problem, we propose Sceptic, a support vector machine model for supervised pseudotime analysis. We demonstrate that Sceptic achieves significantly improved prediction power relative to state-of-the-art methods, and that Sceptic can be applied to a variety of single-cell data types, including single-nucleus image data.

**Supplementary information:**

The online version contains supplementary material available at 10.1186/s13059-025-03679-3.

## Background

With the decreasing cost of single-cell RNA-sequencing (scRNA-seq), it is increasingly common to collect time-series scRNA-seq data sets. Such data can be used to investigate transcriptional changes over various notions of biological time, such as cell differentiation [1], embryonic development [2], and response to stimulus [3].

However, analysis of any time series single-cell dataset requires that we distinguish between “time” and “pseudotime.” The first of these terms is well-defined: “time” simply refers to the time point at which each sample was collected. “Pseudotime,” on the other hand, refers to a “quantitative measure of progress through a biological process” [4]. The term was introduced in the context of single-cell genomics as a way to segregate a collection of measured cells along a developmental trajectory. The key idea is that measurement of a heterogeneous population of cells collected at a single time point may still capture cells in many different stages of development.

Accordingly, many pseudotime inference procedures have been developed, each attempting to automatically infer pseudotimes for individual cells, frequently with respect to branching trajectories. Principal components analysis (PCA) [5] is commonly regarded as a straightforward and interpretable baseline method for pseudotime inference [6]. Slingshot identifies cell lineages by treating groups of cells as nodes within a graph and identifying a minimum spanning tree connecting these nodes [7]. Monocle 2 [8] and Monocle 3 [9], which have been widely adopted [10], employ distinct underlying algorithms. Monocle 2 utilizes a reversed graph embedding strategy to model cell trajectories, effectively constructing a minimum spanning tree among cells [8]. Monocle 3 identifies cell trajectories using a single-rooted directed acyclic graph, which captures the hierarchical organization of cell states [9]. Finally, Palantir is designed to model the trajectories of differentiating cells by treating the determination of cell fate as a probabilistic process, using entropy as a key metric to quantify cell plasticity as cells progress [11]. Each of these methods is typically applied to data derived from a set of cells collected at a single time point. An alternative means of characterizing cell state trajectories is via RNA velocity, which involves analyzing spliced and unspliced reads from scRNA-seq [12]. However, this approach requires full-length sequencing data with splicing information, which is unavailable in datasets from 3′-end sciRNA-seq or 5′-end 10x Genomics. Our method bypasses splicing, relying only on time-series labels to infer pseudotime trajectories. This makes Sceptic applicable across diverse platforms, offering a complementary approach to RNA velocity.

When single-cell data is collected from multiple time points, we must somehow integrate these two notions of time. At least four different methods for achieving this integration are possible. First, some early time series scRNA-seq studies ignored pseudotime entirely, opting to focus on how gene expression changes as a function of time [13]. Second, one can ignore the time labels by applying a pseudotime inference algorithm to the concatenated dataset. This is the most commonly adopted approach. Third, in principle one could run a pseudotime inference algorithm separately on each time point and then, in a post-processing step, somehow calibrate the pseudotime labels across time points. However, we are not aware of any methods that have attempted this type of post hoc calibration.

**Fig. 1:**
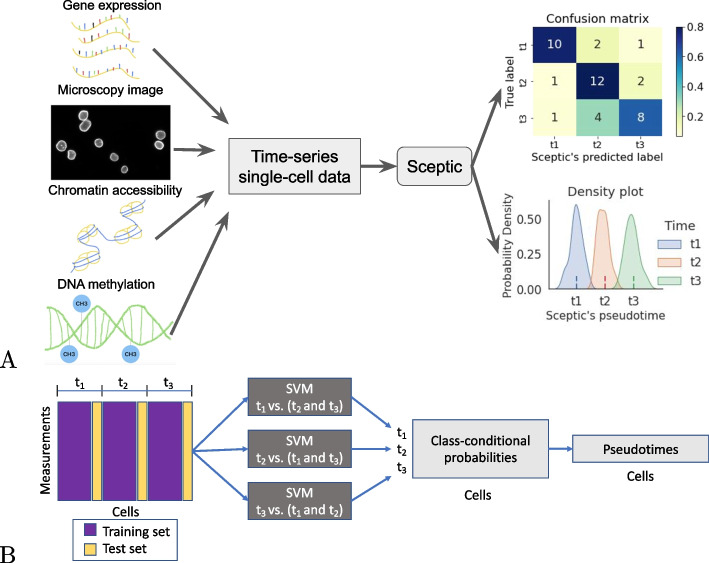
Sceptic analysis for time-series single-cell sequencing and imaging data. **A** Sceptic from the user’s perspective. Sceptic can take various types of single-cell or single-nucleus data as input and is trained to predict each cell’s time label (shown as a confusion matrix) and to infer the pseudotime for each cell (shown as a density plot across all cells). **B** Overview of Sceptic analysis

The fourth approach for integration of time and pseudotime—inferring pseudotimes while taking into account the observed time labels—is the one we pursue here. Several previous methods have adopted related approaches. For example, Tempora focuses on delineating how cell types are interrelated throughout a time series dataset [14]. The method first clusters cells into nominal cell types and infers a trajectory at the cell type level. The directionality of each edge in this cell-type trajectory is then inferred using the associated time labels. Subsequently, psupertime proposed transforming the unsupervised learning problem of assigning pseudotimes to cells into a supervised problem by using the observed times as labels in a regression setup [6]. Their psupertime software uses a penalized ordinal logistical regression model for time-series scRNAseq data. As in several other cases where supervised methods have been used to improve upon unsupervised single-cell analyses [15, 16], psupertime has been shown to perform comparably to or better than competing methods, including PCA, Monocle 2, Slingshot, and Tempora.

We hypothesized that psupertime’s predictive accuracy suffers from its use of a simple linear combination of gene expression profiles. Motivated by this hypothesis, we developed Sceptic, a support vector machine (SVM) framework for supervised pseudotime analysis. Sceptic trains a series of one-vs-the-rest classifiers, thereby generating for each cell a probability vector over all the time points in the dataset. Sceptic then predicts each cell’s pseudotime via conditional expectation. We validated the performance of Sceptic on multiple public single-cell datasets and further extended it to different modalities of time-series single-cell data, including our own single-nucleus imaging dataset. First, we demonstrated that Sceptic achieves higher accuracy than psupertime on six scRNA-seq datasets. Second, we showed that Sceptic works well for single-nucleus imaging data. Third, we applied Sceptic to scATAC-seq data and showed that the model captures sex-specific differentiation from both scATAC-seq and scRNA-seq data. Finally, we applied Sceptic to a co-assay data set, where we detected a methylation delay that agrees with other independent studies. Sceptic’s Python code can be found, with a MIT license, at https://github.com/Noble-Lab/Sceptic.

## Results

### Overview of Sceptic, single cell pseudotime classifier

Sceptic can perform pseudotime analysis on various types of single-cell or single-nucleus data. The model takes as input a collection of such data, learns the relationship between the observed data and the associated time stamps, and finally uses the trained model to assign to each cell a real-valued pseudotime. Ideally, the pseudotimes assigned by Sceptic reflect each cell’s progression along a notion of time—developmental, cell cycle, disease progression, and aging—that is appropriate to the given data.

Sceptic differs from its predecessor, psupertime [6], in three ways (Fig. 1). First, Sceptic uses a nonlinear support vector machine rather than an ordinal logistic regression model. Second, and more importantly, whereas psupertime trains a single regressor with multiple thresholds, Sceptic trains a collection of classifiers, one for each timestamp, using a one-versus-the-rest strategy. The final pseudotime assigned to a given cell is computed as a weighted sum of the different predictions. This setup significantly enhances the classifier’s performance. Third, Sceptic employs a standard cross-validation strategy, in which a collection of models is trained on different subsets of the data and used to make predictions on corresponding test sets. This strategy prevents the model from reporting pseudotime values that may overfit the data.

**Fig. 2:**
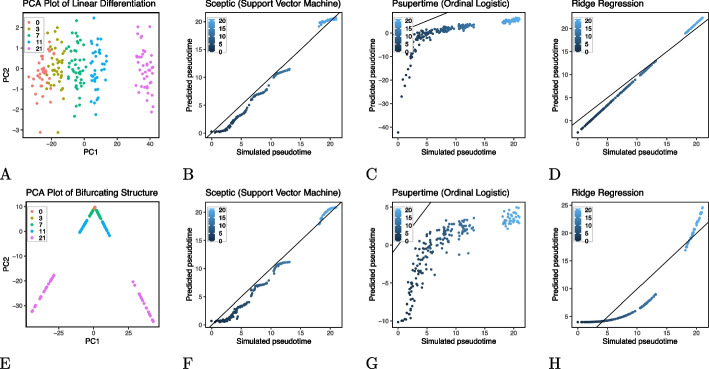
Sceptic works well in simulation. **A** PCA visualization of linear differentiation data. **B** Sceptic’s performance for linear differentiation data. **C** Psupertime’s performance for linear differentiation data. **D** Ridge regression’s performance for linear differentiation data. **E** PCA visualization of bifurcating structure data. **F** Sceptic’s performance for bifurcating structure data. **G** Puspertime’s performance for bifurcating structure data. **H** Ridge regression’s performance for bifurcating structure data

As motivation, we created two simple simulations to illustrate how Sceptic works (details in “Simulation” section). The simulation framework is modeled after an existing collection of mouse embryonic stem cell (mESC) data [1], and the simulation approach is inspired by the one employed by dynverse [10]. We simulated two scenarios: linear differentiation and a bifurcating structure. In each scenario, we compared Sceptic with psupertime and a ridge regression baseline. The results (Fig. 2) suggest that, in the linear differentiation setting, all three methods accurately preserve the simulated ordering of cells; however, only Sceptic and ridge regression accurately predict the true pseudotime values. Psupertime’s predictions, in contrast, are a monotonic transformation of the true pseudotime. In the bifurcating structure setting, Sceptic achieves the best prediction among all three methods by not only preserving the ordering of cells but also predicting the correct scaling of the pseudotimes. Psupertime fails to predict the correct ordering of cells, and ridge regression’s predictions do not reflect the actual scale of the simulated pseudotimes.

To further enhance the simulation, we adopt the framework in psupertime, where we incorporated global time series, cell type time series, batch effects, and non-specific genes (see the descriptions in Section 2.6 of [6]). Our results show that Sceptic outperforms or is comparable to psupertime across 20 different randomly generated datasets (Additional file 1: Fig. S1). Moreover, to evaluate Sceptic’s robustness in more realistic single-cell RNA-seq scenarios, we conducted additional simulations incorporating varying dropout rates (Additional file 1: Fig. S2) and noise levels (various noise magnitudes: Additional file 1: Fig. S3; various numbers of irrelevant genes: Additional file 1: Fig. S4).

### Sceptic outperforms psupertime on classification for time-series single-cell RNA-sequencing data

We designed Sceptic to provide a flexible framework for modeling pseudotime trends within a collection of single-cell data. Accordingly, we set out to test the hypothesis that Sceptic could more accurately predict the timestamp associated with a given cell than psupertime. To do this, we used our recently published scRNA-seq data from a mouse embryonic stem cell (mESC) differentiating time-series dataset, collected over five time points: days 0, 3, 7, 11 and neural progenitor cells collected at day 21 [1]. We used five-fold cross-validation, training a model on 80% of the cells and testing on the remaining 20%, and repeating this procedure five times. For comparison, we also applied psupertime to the same dataset. Because psupertime is not designed to generalize to new cells, we are only able to report psupertime’s performance when fitting a single model to the entire dataset. In this sense, the comparison is biased in favor of psupertime.

**Fig. 3:**
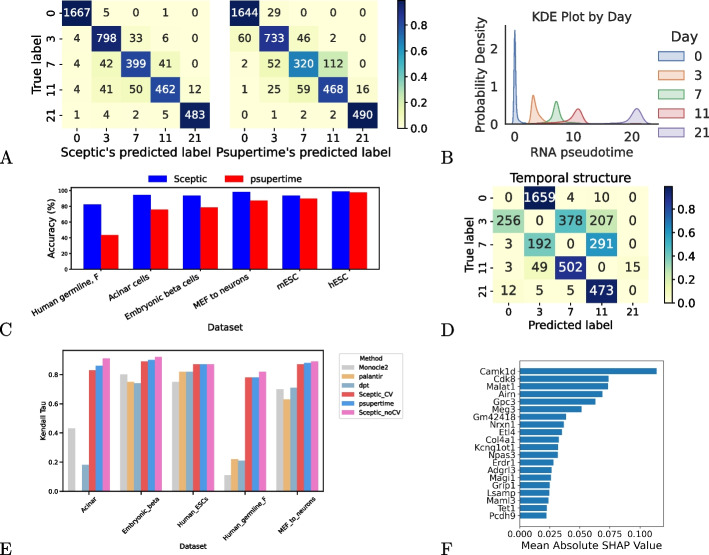
Sceptic achieves better classification accuracy than psupertime on single-cell RNA sequencing data. **A** Confusion matrices for Sceptic (left) and psupertime (right) on the mESC data. Sceptic results use cross-validation, whereas psupertime results are from training on the full dataset. **B** Kernel density plot of Sceptic pseudotime, colored by the day of cells, on the mESC data. **C** Accuracy of both methods on six time-series datasets. Note that psupertime has previously been shown to perform comparably to or better than other methods—including Monocle 2, slingshot, and Tempora—on five of these datasets. Thus, we only compare our performance with psupertime. **D** Confusion matrix from leave-one-time-point-out analysis. A successful model would tend to predict time points adjacent to the diagonal. **E** Comparison of performance using Kendall $\tau$. Sceptic with cross-validation (Sceptic_CV) outperforms or is comparable to most unsupervised methods. Sceptic without cross-validation (Sceptic_noCV) achieves a higher Kendall $\tau$ coeffecient than psupertime. **F** Top 20 genes influencing prediction on mESC data, selected via mean absolute SHAP values

Despite this bias, the predictions from the two models, summarized in confusion matrices (Fig. 3A), show that Sceptic outperforms psupertime. In particular, Sceptic achieves an accuracy of 93.73% (because 3809 of the 4064 predictions fall along the diagonal of the confusion matrix), whereas psupertime’s accuracy is only 89.94% (3655 correct predictions). This difference is significant ($p={4.94e^{-10}}$) according to Fisher’s exact test. The confusion matrices indicate which timestamps are difficult to differentiate, with most of the off-diagonal predictions occurring between days 3 and 7 and between days 7 and 11. Sceptic achieves a clear separation between cells of days 7 and 11 (Fig. 3B), whereas psupertime does not perform very well on these cells (Fig. 3A).

To further assess Sceptic’s performance, we carried out two ablation experiments. First, to assess the impact of allowing non-linear decision boundaries, we implemented a version of Sceptic using an SVM model with only linear kernels. This version showed a performance drop of 1.5% in terms of both accuracy compared to the full version of Sceptic (Additional file 1: Fig. S5). Second, we evaluated the role of nested cross-validation by comparing the performance of Sceptic with and without this feature. As shown in Fig. 3E, the version without cross-validation consistently underperforms the version with nested cross-validation, as expected. Thus, by incorporating nested cross-validation, Sceptic prevents overfitting on the training data and achieves more reliable performance on unseen data.

To further test our hypothesis, we downloaded the five datasets that were analyzed in the original psupertime paper [6], and we repeated the above analysis. For psupertime’s performance, we use the previously reported accuracy and Kendall $\tau$ values. Note that psupertime has previously been shown to perform comparably to or better than other methods, including PCA [5], Monocle 2 [8], Slingshot [7], and Tempora [14], on these five datasets. Again, despite the bias in favor of psupertime in this setup, on all five datasets Sceptic achieves consistently higher classification accuracy than psupertime (Fig. 3C), improving by 1.4%–39.0%. The difference is significant ($p=0.03125$) according to a Wilcoxon signed-rank test.

We further evaluated Sceptic’s performance using Kendall $\tau$ coefficient, comparing with psupertime and three state-of-the-art supervised methods, DPT [17], Palantir [11], and Monocle 2 [8]. In our added benchmarking results (Fig. 3E), we found that the two supervised methods, psupertime and Sceptic, consistently outperform all three unsupervised pseudotime inference methods. In this analysis, we included two variants of Sceptic, one that includes the cross-validation procedure (Sceptic_CV) and one without cross-validation (Sceptic_noCV). The latter is included only to enable a direct comparison with psupertime, which also leaves off the cross-validation step. We observe that Sceptic without cross-validation (Sceptic_noCV) achieves a higher Kendall $\tau$ coefficient than psupertime (Fig. 3E). The fact that Sceptic_noCV outperforms Sceptic_CV is attributable to the fact that the former is effectively being evaluated on the same data that was used to train the model. These results highlight the advantages of Sceptic’s pseudotime in leveraging supervised learning for pseudotime estimation.

We note that, in any evaluation of a supervised pseudotime inference method, we face the fundamental challenge of distinguishing between batch effects and true pseudotemporal ordering. To understand the problem, consider a theoretical dataset in which each time point is coupled with a very strong, time-specific batch effect. In such a setting, a method that successfully learns to identify this batch effect will perform perfectly in a confusion matrix, despite not having learned anything about the temporal signal in the data. Therefore, additional validation is required to demonstrate that the pseudotemporal ordering is meaningful.

To address this concern, we repeated our analysis but used a cross-validation strategy in which we iteratively left one time point out of the training set on mESC dataset. In this setting, the resulting confusion matrix (Fig. 3D) necessarily has zeroes on the diagonal. Our performance measure is the proportion of values that fall in boxes that are adjacent to the diagonal. The reasoning here is that if the model learned only batch effects, then the errors would be uniformly distributed across all time points. On the other hand, a model that successfully learns temporal patterns will, when forced to predict a time for a novel time point, tend to predict adjacent time points. In our case, we find that 92.7% of the predictions in the leave-one-time-point-out setting fall into adjacent time points. This is substantially greater than the 40.0% that would be expected if the errors were uniformly distributed over time points. Note that generalizing this experiment to a leave-two-time-points-out setting would be challenging, because this datset only has five time points in total.

To identify the genes that are important for Sceptic’s predictions, we calculated SHAP (SHapley Additive exPlanations) values [18] to evaluate feature importance (Fig. 3F). By computing the mean absolute SHAP value across different categories, we identified the top 20 genes that contribute most to prediction performance. We analyzed the association of each of these genes with Gene Ontology annotations and the imprinting database (Additional file 2: Table S1) and identifed several trends. Notably, the genes *Airn*, *Meg3*, and *Kcnq1ot1* are well known imprinting genes [19], which suggests a connection to genomic imprinting processes during mouse embryonic differentiation; *Nrxn1* [20], *Npas3* [21], *Adgrl3* [22], and *Grip1* [23] are strongly associated with synaptic function and brain development; *Tet1* and *Meg3* are involved in epigenetic modifications during development. These findings not only provide biological insights into the genes influencing classification but also suggest potential links between genomic imprinting, neurodevelopmental processes, and epigenetic regulation during embryonic differentiation.

**Fig. 4:**
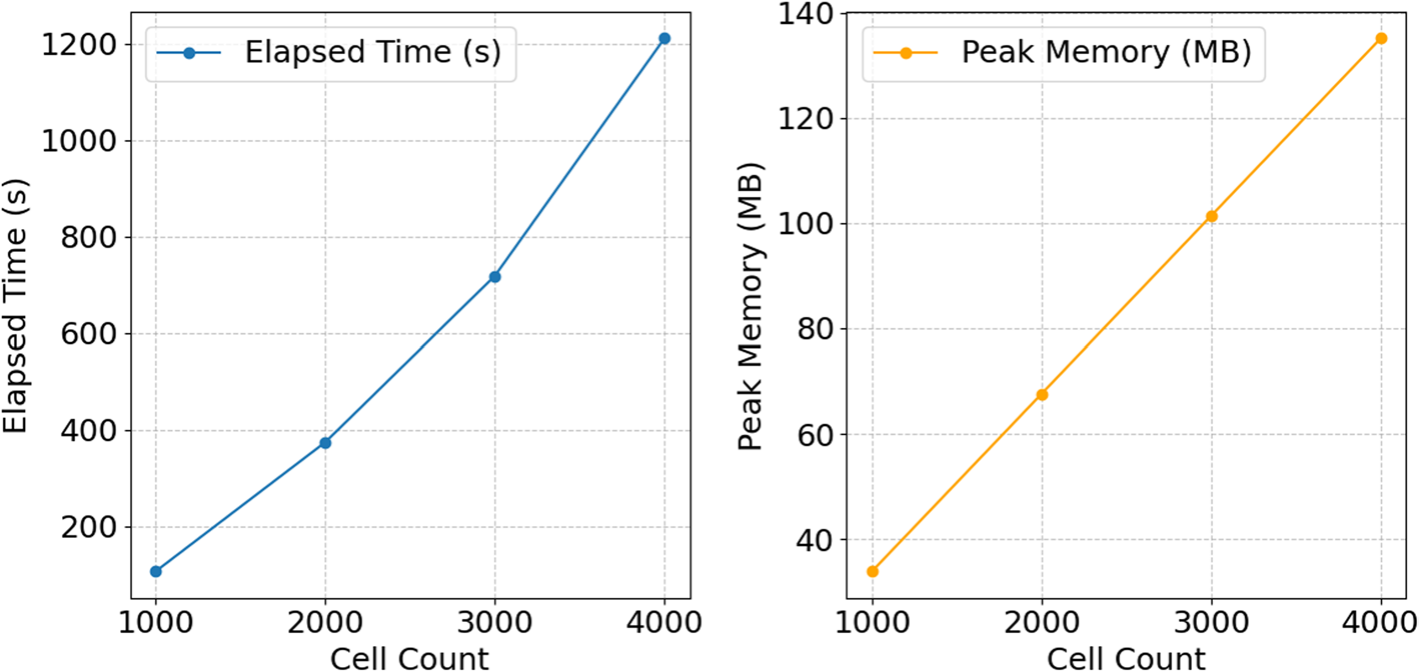
Time and RAM consumption of Sceptic

We also evaluated Sceptic’s time and RAM consumption for datasets of varying sizes, ranging from 1000 to 4000 cells. These results provide insights into Sceptic’s computational efficiency on datasets of practical sizes. The results (Fig. 4) show that Sceptic’s resource requirements increase linearly with the number of cells. This analysis highlights the strengths and current limitations of Sceptic while providing a foundation for future work aimed at improving scalability.

### Sceptic works well for single-nucleus imaging data

**Fig. 5:**
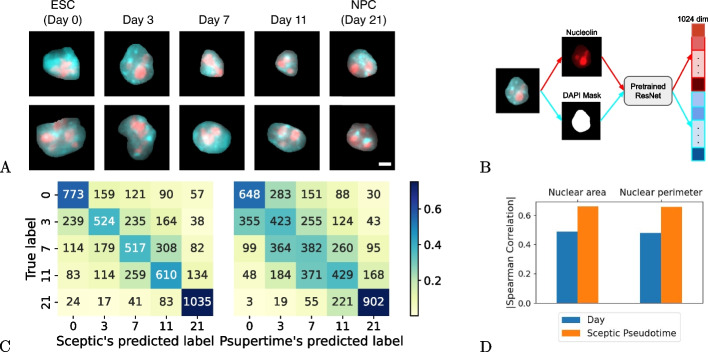
Sceptic works well for single-nucleus imaging data. **A** Examples of single nucleus images. Blue = DAPI; red = nucleolin. Scale bar is 10 $\mu$m. ESC: embryonic stem cells. NPC: neural progenitor cells. **B** Image processing pipeline. **C** Sceptic achieves better classification accuracy than psupertime for time-series single-nucleus image data. **D** Pseudotimes inferred by Sceptic are more highly correlated with nuclear morphology features than the observed time label

Having established that Sceptic’s machine learning framework works well for scRNA-seq data, we next investigated whether a similar approach would generalize to single-nucleus microscopy imaging data. Accordingly, we collected F121-6 mESC differentiation 3D images (DNA staining with DAPI and immunostaining of nucleolin) using the same five time points as described in our recent study [1] (Fig. 5A). We segmented each 2D-maximum-projection image using a pre-trained Mask R-CNN model [24, 25], and we used a pre-trained ResNet34 model to generate 1024-dimensional representations for each nucleus (Fig. 5B). To measure the performance of the classifier, we randomly selected 1200 single-nucleus image crops from each time point, yielding 6000 crops in total.

Our results show that Sceptic is able to accurately assign time labels to nuclei on the basis of microscopy images, significantly outperforming psupertime on this task (Fig. 5C). Overall, Sceptic achieves 57.7% test set accuracy, whereas psupertime achieves a significantly lower 46.4% training set accuracy ($p={1.08e^{-8}}$, Fisher’s exact test). For both methods, the overall accuracy is lower in the microscopy setting than in the scRNA-seq setting. As expected, Sceptic achieves the best separation between the ends of the time series—day 0 versus day 21.

Next we investigated whether the time labels assigned by Sceptic show stronger correlations with cell morphology features than the observed time labels. During embryonic stem cell differentiation, neural progenitor cells (day 21) exhibit reduced nucleolus activity, reduced transcription of rRNA genes, and smaller nucleoli [26]. Thus, we expect an accurate time label to be strongly correlated with these morphology features. We therefore compared the correlations between pseudotime or observed time with respect to two features: nuclear area and nuclear perimeter. Compared to the observed time points, Sceptic assigns much finer-grained continuous time labels, and thus achieves a higher absolute correlation with nuclear image morphology features compared with the discrete-time labels (Fig. 5D). These results suggest that the pseudotime values inferred by Sceptic accurately capture the heterogeneity of nuclei sampled at the same time point and thus provide more informative time labels than the observed ones.

### Sceptic detects sex-specific differentiation from scATAC-seq and scRNA-seq data

Having established that Sceptic can work well for both scRNA-seq and single-nucleus imaging data, we next investigated whether a similar approach could generalize to single-cell transposase-accessible chromatin with sequencing (scATAC-seq) data. This assay identifies, in a cell-specific fashion, punctate regions of open chromatin, which typically correspond to various types of regulatory elements, including promoters, enhancers, and insulators. To test Sceptic, we downloaded F121-6 (female) and F123 (male) mESC differentiation scATAC-seq data from Bonora et al. [1]. To address the issue of imbalance in the number of cells across time points, we randomly downsampled the number of cells from each time point to the smallest cell number across all time points, which left us with 535 female cells (107 cells for each time point) and 560 male cells (140 cells for each time point). We trained two separate Sceptic models for each dataset, one for each sex.

**Fig. 6:**
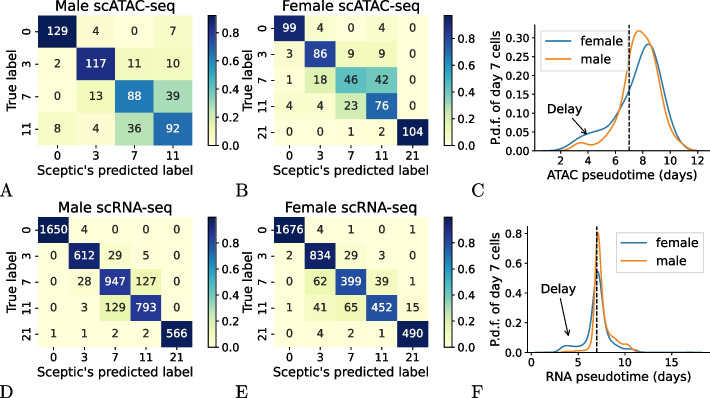
Sceptic applied to scATAC-seq and scRNA-seq mESC data. **A** Sceptic’s performance for scATAC-seq data of F123 (male) cells. **B** Sceptic’s performance for scATAC-seq data of F121-6 (female) cells. **C** Probability density function of Sceptic’s pseudotime for day 7 cells of scATAC-seq data. **D** Sceptic’s performance for scRNA-seq data of F123 (male) cells. **E** Sceptic’s performance for scRNA-seq data of F121-6 (female) cells. **F** Probability density function of Sceptic’s pseudotime for day 7 cells of scRNA-seq data

In this experiment, Sceptic achieves 76.8% test set accuracy on F121-6 female cells and 76.1% on F123 male cells (Fig. 6A–B). We note that Sceptic accurately classifies cells from day 0 and day 3 for both male and female cells, whereas, on both datasets, cells from days 7 and 11 are difficult to distinguish. Cells from day 7 and day 11 share similar chromatin accessibility patterns, and they construct a single cell trajectory branch (embryonic body branch) in Bonora et al.’s analyses. In addition, we observed that for day 7, female cells have a slightly higher proportion of cells classified as earlier time points (day 0 and day 3) than their male counterparts (Fig. 6A–C): 17.8% (19 out of 107 cells) for female and 9.3% (13 out of 140 cells) for male. Similar differences in proportion are observed for cells in day 11 (34.3% for female cells and 29.0% for male cells). These differences are potentially indicative of female-delayed onset of differentiation and are consistent with previous observations [1, 27, 28], in which the early establishment of X chromosome inactivation is required for differentiation.

To follow up on this observation, we next use Sceptic to infer pseudotimes from scRNA-seq data generated from the same series of mESC samples [1]. For this data, Sceptic achieves an accuracy of $93.2\%$ for male and $93.4\%$ for female across five time points (Fig. 6D–E). As in the ATAC-seq setting, we found for day 7 that female cells have a higher proportion of cells classified as earlier time points (12.4%; 62 out of 500 cells) than their male counterparts (2.5%; 28 out of 1102 cells) (Fig. 6D–F), and similar proportions are observed for cells in day 11 (18.6% for female cells and 14.3% for male cells). This is consistent with our observation on scATAC-seq, but we found the transcript delay pattern is much stronger than the delay of chromatin accessibility. In addition, we compared Sceptic’s performance with psupertime on the scATAC-seq data (Additional file 1: Fig. S6), where Sceptic shows uniformly better classification accuracy on both male and female cells.

When comparing Sceptic’s performance across modalities, we found that Sceptic achieves better classification accuracy with scRNA-seq data compared to scATAC-seq data (Additional file 1: Fig. S7). We speculate that this difference arises for two reasons. First, scRNA-seq directly captures transcriptomic states, which provide clear and biologically meaningful signals of cellular transitions. In contrast, chromatin accessibility data from scATAC-seq is sparser and thus noisier [19, 29], and is usually binarized to be closed or open, yielding a more limited dynamic range [30]. In many cases, an open chromatin region may remain open even when the genes it regulates are not expressed. For example, in embryonic stem cells, poised enhancers are associated with genes involved in lineage-specific differentiation. While they remain open and accessible but do not drive gene expression until the cell receives signals to differentiate into a specific cell type [31]. Second, scATAC-seq data is inherently sparser, with a significantly larger feature space compared to scRNA-seq (>200,000 peaks versus $\sim$20,000 genes). This increased sparsity and dimensionality make it more challenging for models to extract meaningful patterns for classification.

For scATAC-seq analysis, we compared Sceptic with Cicero—a Monocle-based framework [32] for pseudotime inference—using both male and female mESC data. We found that Cicero requires manual selection of the root cell and often assigns infinite pseudotime values to cells positioned far from the starting point in the trajectory, reducing its robustness for this type of analysis. As shown in Additional file 1: Fig. S8, our Sceptic model consistently outperforms Cicero, achieving a higher mean absolute Kendall tau coefficient by 0.22 to 0.31 for both male and female datasets.

### Sceptic identifies methylation delay using co-assay data

We also applied our Sceptic model to a co-assay data, sc-GEM, a protocol that simultaneously measure pre-selected DNA methylation and gene expression markers in single cells via polymerase chain reaction (PCR) [33]. Cheow et al. performed sc-GEM on human fibroblasts undergoing induced pluripotent stem (iPS) cell reprogramming. This dataset contains 177 cells from five time points, BJ (day 0; fibroblasts established from skin taken from normal foreskin from a neonatal male), day 8, day 16, day 24, and iPS.

**Fig. 7:**
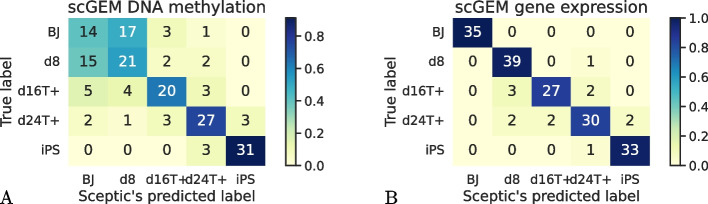
Sceptic captures DNA methylation delay. **A** Sceptic can also separate cells using DNA methylation data, albeit with some ambiguity between day 0 (BJ) and day 8. **B** Sceptic can accurately distinguish cells from 5 times points using gene expression data

For this data set, Sceptic achieves 92.7% test set accuracy using gene expression data but only 63.8% using DNA methylation data (Fig. 7). In particular, using methylation data, Sceptic can accurately distinguish among the later three time points well but struggles to distinguish between day 0 and day 8 cells. This observation suggests that there are enough transcriptomic changes within 8 days of the reprogramming process to distinguish untreated cells and day 8 cells, but it takes longer than 8 days to distinguish DNA methylation state changes. Sceptic’s results are consistent with a previous study, which observed that most of the DNA methylation changes in iPS reprogramming only occur after day 9 [34].

We explored the concordance between pseudotimes inferred from different modalities. To investigate this, we performed a two-way analysis of variance (ANOVA) to evaluate the influence of RNA-inferred pseudotime and time on methylation pseudotime. Notably, the analysis revealed a significant impact of RNA pseudotime on the variation in methylation pseudotime (*P *value = 4.20e−11). This finding implies that the pseudotimes generated by two Sceptic models capture common underlying structures within the same cells, highlighting their shared aspects.

We also investigated Sceptic’s ability to work with single-cell Hi-C data, using allelic embryonic stem cell scHi-C data from a recent study [1]. Across five time points, Sceptic achieves 87.47% test set accuracy and outperforms psupertime by a large margin. (Additional file 1: Fig. S9).

### Scaling up Sceptic to large datasets

To enhance computational efficiency and scalability, we implemented an option in Sceptic to use XGBoost [35] with GPU acceleration as an alternative to the default SVM classifier. The XGBoost implementation takes the same cell-by-measurement matrix as input and outputs cell-specific class probabilities. These probabilities are then used to compute pseudotime values via the same conditional expectation framework applied in the original SVM-based method.

**Fig. 8:**
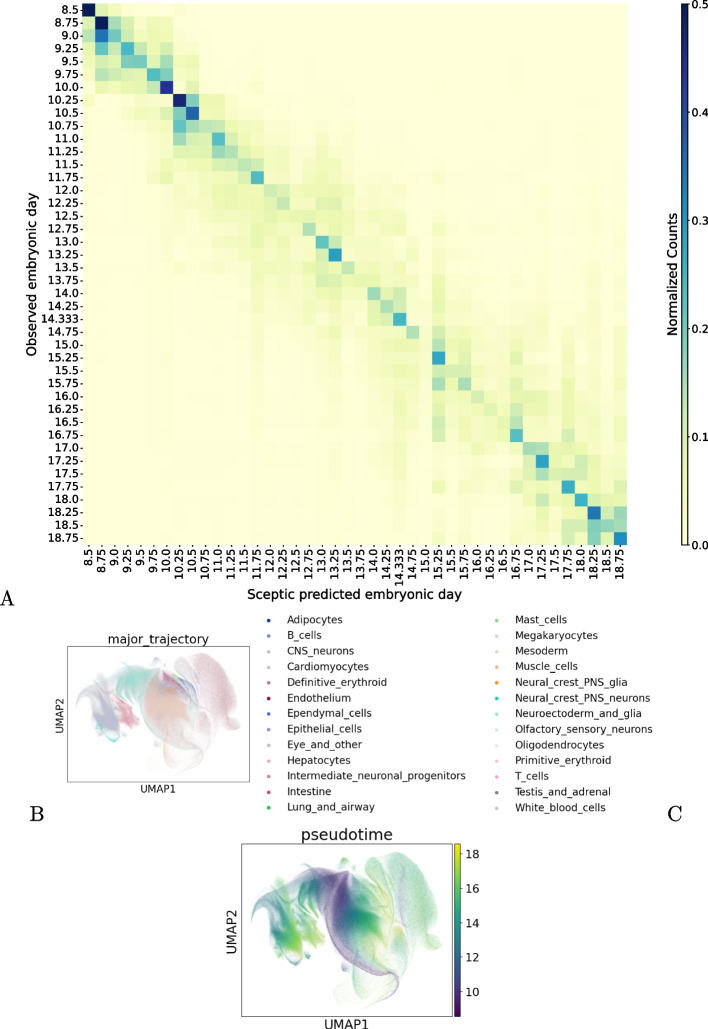
Sceptic applied to an atlas of mouse embryogenesis. **A** Confusion matrix for Sceptic on the mouse embryogenesis atlas data. The color scale is the row-normalized cell counts and it is truncated at a maximum value of 0.5 to enhance contrast. **B** UMAP of mouse embryogenesis cells, colored by cell type. **C** UMAP of mouse embryogenesis cells, colored by Sceptic’s inferred pseudotime

To demonstrate the scalability of the enhanced Sceptic model, we performed pseudotime analysis on the mouse embryonic development dataset [36]. This dataset comprises single-nucleus transcriptomes from 12.4 million cells across 83 precisely staged mouse embryos, capturing the full course of prenatal development from late gastrulation (E8) to birth at high temporal resolution. To balance the number of cells across different time points, we downsampled the oversampled stages to match the time point with the fewest cells. Specifically, we randomly selected 46,253 cells from each of the 42 embryonic stages (excluding P0) and used the resulting balanced dataset (1.9 million cells in total) for pseudotime inference. We found that Sceptic accurately positions most cells near the diagonal in the pseudotime versus observed time plot, reflecting the temporal proximity of neighboring stages (Fig. 8A). This result highlights Sceptic’s ability to preserve fine-grained temporal structure, even when the true time labels are closely spaced. We also generated a UMAP embedding colored by Sceptic-inferred pseudotime, which clearly illustrates the continuous temporal progression of cells along the trajectory (Fig. 8B–C). Together, these results demonstrate Sceptic’s ability to scale to large, multi-cell-type datasets and its potential to generalize across complex time-series single-cell data.

## Discussion

In this work, we developed a method, Sceptic, to infer pseudotime for single-cell sequencing and imaging data using a supervised machine learning approach. Our experiments suggest that Sceptic offers improved accuracy relative to the previous supervised method, psupertime. Our extensive evaluation demonstrates that Sceptic effectively infers pseudotime for time-series sequencing and imaging data. Application of Sceptic to multiple single-cell RNA-sequencing data has shown uniformly improved performance compared with state-of-the-art methods. The extension to scATAC-seq and single nucleus imaging is straightforward and effective, and it successfully captures the cells in the same cell trajectory but from different time points and the cell heterogeneity within the same time points, providing a better time label for each cell.

There are several potential avenues for improvements to Sceptic. First, whereas psupertime’s linear model is relatively easy to interpret, Sceptic’s non-linear kernel functions make such interpretation more challenging. Intuitively, for more complex data structures like imaging, it is natural to use non-linear decision boundaries for high-dimensional classification problems. But it would still be interesting to find which genes are significantly important for Sceptic’s classifiers. Permutation tests could be an effective option when the number of genes and the number of cells are not too large.

Second, our current Sceptic model has been shown to work well for a simple cell differentiation trajectory or reprogramming process. However, one might consider how to compare pseudotimes across multiple concurrent lineages or cell trajectories.

Finally, when the sampling time points are very dense (say, sequencing every 6 h) and the number of time points is large (say, greater than 40), then one might also consider formulating the pseudotime analysis problem directly as a regression problem. Along these lines, Calderon et al. adopted a neural network as a regression tool to infer pseudotime for *Drosophila* embryonic development [37]. Because their dataset was collected at overlapping time windows and we mainly focused on classification evaluation, we did not evaluate this dataset. It would be great to combine both the classification and regression nature of pseudotime analysis into a single loss function.

## Conclusions

In this study, we introduced Sceptic, a supervised machine learning method for pseudotime inference from single-cell data. Relative to existing approaches, Sceptic demonstrates substantially improved predictive accuracy across diverse data types, including time-series scRNA-seq, scATAC-seq, and single-nucleus imaging data. By effectively capturing both global developmental trajectories and local heterogeneity, Sceptic provides more accurate and generalizable temporal labels for single-cell analyses. In the future, extending Sceptic to handle branching trajectories and formulating hybrid models that unify classification and regression objectives could further enhance its applicability to high-resolution developmental time courses. Overall, Sceptic represents a robust and versatile framework for supervised pseudotime inference, offering a significant step forward in the analysis of dynamic biological processes from single-cell data.

## Methods

### Single cell pseudotime classifier (Sceptic)

Sceptic is an SVM-based model for pseudotime analysis. It takes as input a single-cell measurement matrix *X*, with *M* rows of measurements and *N* columns corresponding to cells, and a corresponding time label vector $\vec {t}=(t_1,t_2, ..., t_N)$. We assume that the cells are collected from *D* different observed time points, i.e., $t_i \in \{T_1, T_2, ..., T_D\}$.

We used SVM models to first estimate the probability of a cell belonging to each discrete time point $T_d$ given its observed gene expression profile. These probabilities are then used to compute the conditional expectation, which reflects the cell’s pseudotime as a weighted sum of the time points.

The probabilities $P(Y | X = x)$ are defined as follows:

$$\begin{aligned} P(Y | X = x) = \left\{ \begin{array}{ll} p_1 & \text {if cell is collected from } T_1, \\ p_2 & \text {if cell is collected from } T_2, \\ \vdots & \vdots \\ p_D & \text {if cell is collected from } T_D. \end{array}\right. \end{aligned}$$

Using these probabilities, the pseudotime is calculated as:

$$\begin{aligned} E(Y|X=x) = \sum \limits _{d=1}^D Prob(\text {cell is collected from } T_d | X=x) \times T_d = \sum \limits _{d=1}^D p_d \times T_d . \end{aligned}$$

This framework ensures that the model accounts for heterogeneity within each time point by assigning a pseudotime to each cell based on the conditional expectation of its time point assignment.

Sceptic trains one support vector machine classifier for each timestamp, say $T_1$, optimizing a coefficient vector $\textbf{a}=(a_1,a_2, ..., a_N)$ for $T_1$ to minimize the following loss with $t_i^{(1)} = +1$ if $t_i = T_1$ and $t_i^{(1)} = -1$ if $t_i \ne T_1$ :

1 $$\begin{aligned} L(\textbf{a})= &\ \sum \limits _{i=1}^N a_i-\frac{1}{2} \sum \limits _{i=1}^N \sum \limits _{j=1}^N a_i a_j t_i^{(1)} t_j^{(1)} K\left( \textbf{x}_i, \textbf{x}_j\right) \nonumber \\ \text {subject to } 0 \le a_i\le &\ C, \quad i=1, \ldots , N \nonumber \\ \sum \limits _{i=1}^N a_i t_i^{(1)}= &\ 0 . \end{aligned}$$

where $K\left( x_i, x_j\right) =\phi \left( x_i\right) ^T \phi \left( x_j\right)$ is the kernel function. Sceptic default uses a radial basis function (RBF) kernel $K\left( x_i, x_j\right) = \exp \left( -\gamma \left\| x_i-x_j\right\| ^2\right)$ but also allows other kernels such as a linear kernel $K\left( x_i, x_j\right) =x_i^Tx_j$. Once fitted, Sceptic estimates a class membership probability ${\textbf {P}}=(p_1,p_2, ..., p_D)$ where $p_d$ is the probability that the cell is collected from time point $T_d$ based on Wu et al. [38]. The pairwise estimated probabilities between any pair of classes are first calibrated using Platt scaling [39] and then used to solve a quadratic optimization where the solution is the class membership probability.

#### Nested cross validation

To select hyperparameters while avoiding overfitting, we train Sceptic using a nested cross-validation scheme. Cross-validation involves randomly splitting the data into, say, five equal-sized folds, and then iteratively using four folds to train the model and the remaining fold for testing. Nested cross-validation repeats another cross-validation splitting procedure on each training set. We use the internal 4-fold cross-validation step to select a set of optimal hyperparameters for each training set, and these hyperparameters are then used to train a model on the full training set. The trained model is then used to make predictions on the test set. We repeat this process five times as the external 5-fold cross-validation step to make predictions on all the data.

#### Hyperparameter search

In the inner loop of the nested cross-validation, Sceptic performs a grid search over three hyperparameters. The first hyperparameter *R* is a Boolean indicating whether the SVM uses a linear or RBF kernel. The second hyperparameter *C* controls the relative weight assigned to violations of the SVM soft margin. The third hyperparameter $\gamma$ specifies the inverse of the width of the RBF kernel. We adopt sklearn’s default $\gamma$ for support vector classifier: $\gamma = 1 / (M * Var(vector(X))$, which adjusts the number of features *M* and the variability of the measurement matrix via the variance of the measurement matrix *X* in vector format. Sceptic employs a six-valued grid with dimensions $R \in \{0, 1\}$, $C \in \{0.1, 1, 10\}$.

### Psupertime

For five datasets that psupertime evaluated, we directly compared Sceptic’s results with the reported performance in the psupertime paper. For mESC data, we follow their tutorial using the default setting to select the hyperparameter $\lambda$ for the weight of the L1 regularization penalty, selecting the $\lambda .1se$ with performance within one standard error of the best performance $\lambda .min$, and then re-training the model using all the cells.

### Imaging data

#### Sample collection

F121-6 mouse embryonic stem cells (ESCs) were grown and differentiated into embryoid bodies (EBs) as described in Bonora et al. [1]. Neural progenitor cells (NPCs) were derived from EBs at day 11 and collected at day 21. ESCs and EBs at days 3, 7, and 11, as well as NPCs, were collected and frozen in 10% DMSO in 2–3 million cell aliquots.

#### Immunostaining

Frozen cell aliquots were thawed at 37 C and resuspended in 1x DPBS. Approximately 250,000 cells were plated on a 24-well glass bottom, black-walled plates (CellVis, Mountain View, CA, P24-1.5H-N) via centrifugation at 300 g for 5 min. The cells were then fixed with 4% paraformaldehyde (Electron Microscopy Sciences Cat. No. 50-980-492) for 15 min at room temperature. After fixation, they were washed three times with 1x PBS and incubated in blocking buffer (3% BSA and 0.25% Tx100 in 1x PBS) for 1 h at room temperature. They were then incubated with primary antibodies (Polyclonal nucleolin antibodies, 1:500, Invitrogen Cat. No.PA5-85972) diluted in blocking buffer for an hour at room temperature. The cells were washed three times with 1x PBS and incubated with secondary antibodies (Donkey anti rabbit Alexa 647, Invitrogen, Cat. No. A-31573) and DAPI diluted in blocking buffer for an hour at room temperature. They were then washed three times with 1x PBS and stored in 600 $\mu$L of 1x PBS.

#### Image acquisition

Image acquisition was performed with an Automated Leica DMi8 inverted microscope with Adaptive Focus technology configured with a 40X 0.95 NA objective. The microscope was illuminated using a six-line Lumencor Spectra X Light Engine LED with Semrock multi-band dichroic filters (Spectra Services, Ontario, NY). The images were captured with a Zyla 4.2 sCMOS camera (Andor, Windsor, CT, USA) and the Piezo-driven stage (Okolab) was used to acquire z-stacks.

#### Image segmentation

Nuclear segmentation was performed with a pre-trained Mask R-CNN model [24, 25] with a nuclear size cutoff of 900. Each nucleus was padded and cropped to a 128$\times$128 pixel image.

#### Image embeddings

For each single-nucleus image crop (128$\times$128) of mESC dataset that we collected, we use pre-trained (on ImageNet [40]) Resnet34 [41] (pytorch/vision:v0.10.0) from Pytorch [42] to generate a less-noisy vector-representation of the image. We generate 512-dim embeddings from the nucleolin-tagged image and 512-dim embeddings from the DAPI mask image. By concatenating them together, we generated a 1024-dim representation for each image crop.

### scATAC-seq data

We followed the epiScanpy pipeline to process the mESC scATAC-seq data [43]. Briefly, we first binarized the cell by peak matrix and identified the most variable peaks. The standard library size normalization and log normalization were performed. Finally, we used the top 50 principal components from normalized data as the input for our Sceptic model.

### scRNA-seq data

We used six publicly available scRNA-seq data sets (Table 1). We downloaded the five scRNA-seq datasets that were previously analyzed by Manair et al. from the R package “psupplementary” (https://github.com/wmacnair/psupplementary). We also downloaded the mESC scRNA-seq and scATAC-seq datasets analyzed by Bonora et al. [1] from https://bitbucket.org/noblelab/mouse-sci-omics/src/master/. We downloaded the processed scGEM dataset from UnionCom’s GitHub page (https://github.com/caokai1073/UnionCom/tree/master/scGEM). For scRNA-seq data, we follow the standard preprocessing pipeline of Scanpy, which includes filtering low-quality cells, log-normalization, and the identification of highly variable genes (default 1000 genes). Note that if fewer than 1000 genes are provided for a given data set, then all the genes are used for pseudotime inference.

**Table 1:** Time-series scRNA-seq datasets

Datasets	Type	Time label	# time points	# cells	Accession
mESC [1]	sciRNA	Embryonic day	5	4064	GSE184554
hESC [44]	scRNA	Embryonic day	5	1529	E-MTAB-3929
Human germline [45]	scRNA	Age (weeks)	12	992	GSE86146
Embryonic beta cells [46]	scRNA	Developmental stage	7	575	GSE87375
Acinar cells [13]	scRNA	Donor age	8	411	GSE81547
MEF to neurons [3]	scRNA	Days since induction	5	315	GSE67310

### Simulation

We generated a random set of 200 cells, each assigned to one of five observed time points: days 0, 3, 7, 11, and 21. For each cell, the simulated pseudotime was obtained by sampling from a Gaussian distribution with a mean corresponding to its observed time point. Then the scale of the pseudotime was normalized to a range between 0 and 21, while preserving the ordering of the cells along the pseudotime continuum. This normalization ensures consistency in the pseudotime scale while maintaining the temporal order of the cells.

We first simulated a linear differentiation process. To do so, we performed a linear projection of the normalized pseudotime onto a 500-dimensional gene expression space. This projection was achieved by multiplying the normalized pseudotime values by 500 random Gaussian projection parameters. The result is a 200-by-500 matrix representing the cell-by-gene expression, where each row corresponds to a cell, and each column corresponds to a gene. The dataset is associated with 200 observed time points, and 200 normalized pseudotime values need to be estimated.

Next, we simulated a bifurcating differentiation process. In this setting, for cells corresponding to days 7, 11, and 21, each cell is randomly assigned to one of two branches with a 0.5 probability. Subsequently, the normalized pseudotime for each branch is projected onto a 500-gene expression space by multiplying it with 500 random Gaussian projection parameters specific to that branch. The cubic power transformation is then applied to all genes to simulate the non-linear relationship between genes and pseudotime. This process results in a 200-by-500 matrix representing cell-by-gene expression. As in the first simulation, the dataset is associated with 200 observed time points, and 200 normalized pseudotime values need to be estimated.

## Supplementary information

Additional file 1. Supplementary materials which include supplementary figures S1–S9.

Additional file 2. Supplementary Table S1.

Additional file 3. Review history.

## Data Availability

The MIT-licensed Sceptic source code is available at GitHub (https://github.com/Noble-Lab/Sceptic) and Zenodo (https://doi.org/10.5281/zenodo.15729886) [47]. Image data generated for this project is available at the 4D Nucleome Consortium data portal (https://data.4dnucleome.org/li_sceptic_mESC_microscope) with accession IDs: embryonic stem cells (day 0): 4DNESGFHRFGV; embryoid body day 3: 4DNESIIXJOYP; embryoid body day 7: 4DNESHZJ83A4; embryoid body day 11: 4DNESR8N1O2P; NPCs: 4DNESLP3I9VG. The processed image embeddings are available at https://doi.org/10.5281/zenodo.15746650 [48]. Five public scRNA-seq datasets used in this article are available from the R package “psupplementary” (https://github.com/wmacnair/psupplementary). All mESC multi-omics datasets associated with Bonora et al. [1] are available at https://bitbucket.org/noblelab/mouse-sci-omics/src/master/. The scGEM dataset is available from UnionCom’s GitHub page (https://github.com/caokai1073/UnionCom/tree/master/scGEM).
