# Supplementary material for: Sceptic: pseudotime analysis for time-series single-cell sequencing and imaging data
Source: Genome Biol. 2025 Jul 17;26:209. doi: 10.1186/s13059-025-03679-3 (PMC12273354; doi:10.1186/s13059-025-03679-3)
Supplement: Supplementary file 1 — Additional file 1. Supplementary materials which include supplementary figures S1–S9. [file 13059_2025_3679_MOESM1_ESM.pdf]

Supplement to "Sceptic: pseudotime analysis for time-series single-cell sequencing and imaging data"

Gang Li[1,2], Hyeon-Jin Kim[1], Sriram Pendyala[1], Ran Zhang[1], Jean-Philippe Vert[3], Christine M. Disteche[4,5], Xinxian Deng[4], Douglas M. Fowler[1,6], and William Stafford Noble[1,7]

[1]Department of Genome Sciences, University of Washington, Seattle 98115, USA
[2]eScience Institute, University of Washington, Seattle 98115, USA
[3]Owkin, Paris 75010, France
[4]Department of Laboratory Medicine and Pathology, University of Washington, Seattle 98115, USA
[5]Department of Medicine, University of Washington, Seattle 98115, USA
[6]Department of Bioengineering, University of Washington, Seattle 98115, USA
[7]Paul G. Allen School of Computer Science and Engineering, University of Washington, Seattle 98115, USA

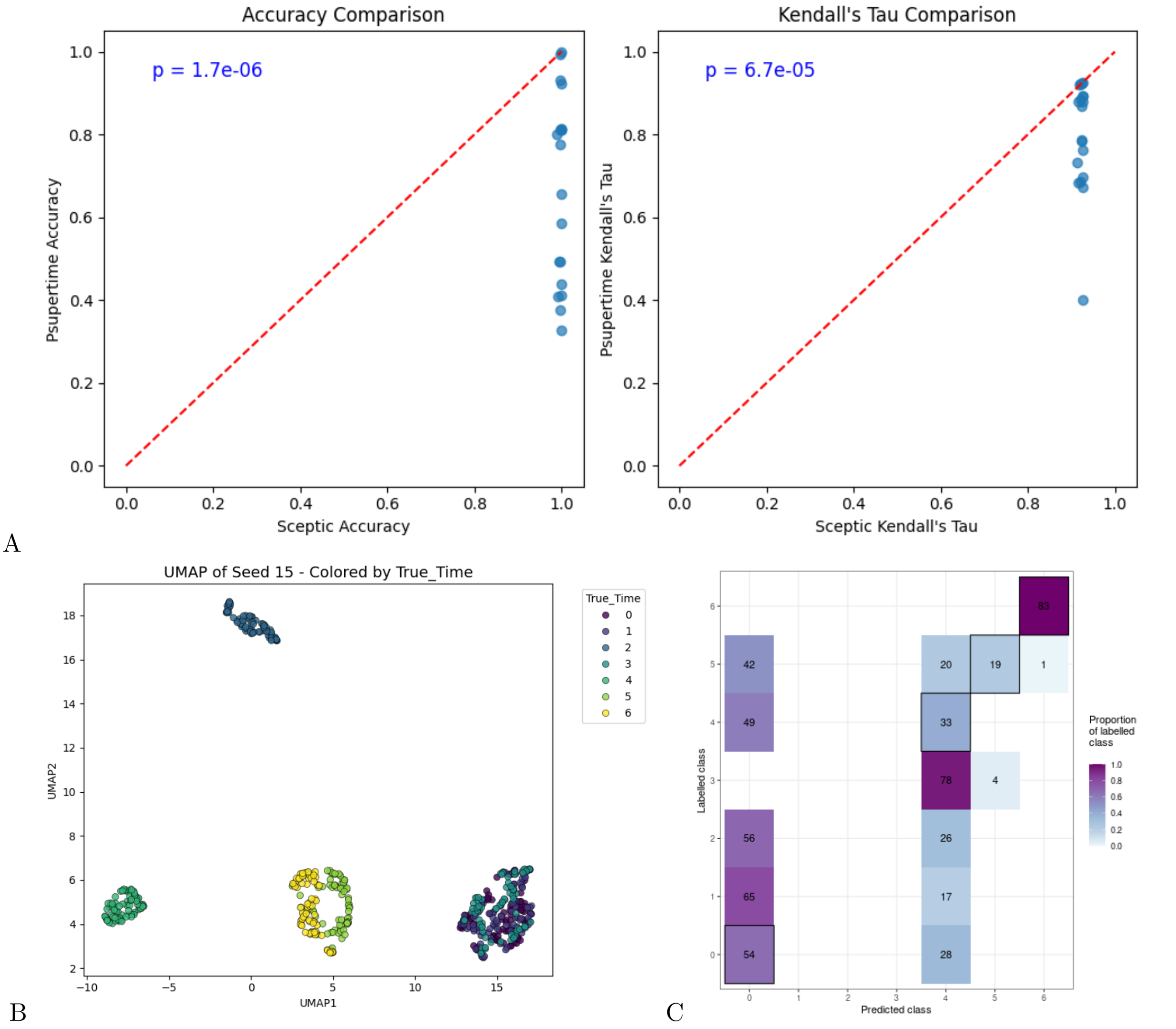

Figure S1: **Comparison of Sceptic and psupertime using the psupertime simulation framework.** **A** Scatter plots of accuracy and Kendall's Tau coefficients. **B-C: Psupertime's prediction on a simulated dataset where it performs the worst.** **B** UMAP of cells colored by the real time. **C** Confusion matrix of Psupertime on the simulated dataset.

To further expand our simulations, we adopted strategies inspired by those used by psupertime. Specifically, we incorporated global time-series trajectories, cell-type-specific time series, batch effects, and the inclusion of non-specific genes into simulation framework, as described by [2]. However, whereas the original psupertime simulation used one of the real datasets as a reference to compute the summary statistics that underlie the simulation, for simplicity we randomly sample two summary statistics, log means and dispersion from a Gaussian distribution (with mean = 1, variance = 0.5) and Gamma distribution (with shape = 2, scale = 0.5), respectively. Under these simulation scenarios, Sceptic consistently demonstrated robust performance, outperforming psupertime for all 20 random seeds across multiple metrics, including classification accuracy and Kendall's τ coefficient. These results further validate Sceptic's ability to handle complex, real-world data.

Each dot represents a simulated dataset generated with a different random seed. We evaluated Sceptic against psupertime based on accuracy and Kendall's tau coefficient. Dots positioned below the diagonal indicate superior performance by Sceptic. A Wilcoxon rank test was used to statistically compare the models across 20 randomly simulated datasets. We further examined psupertime's performance on the random dataset where it performed the worst (Additional File 1: Fig. S1). The simulated data exhibit a non-linear developmental trend, which psupertime fails to capture effectively.

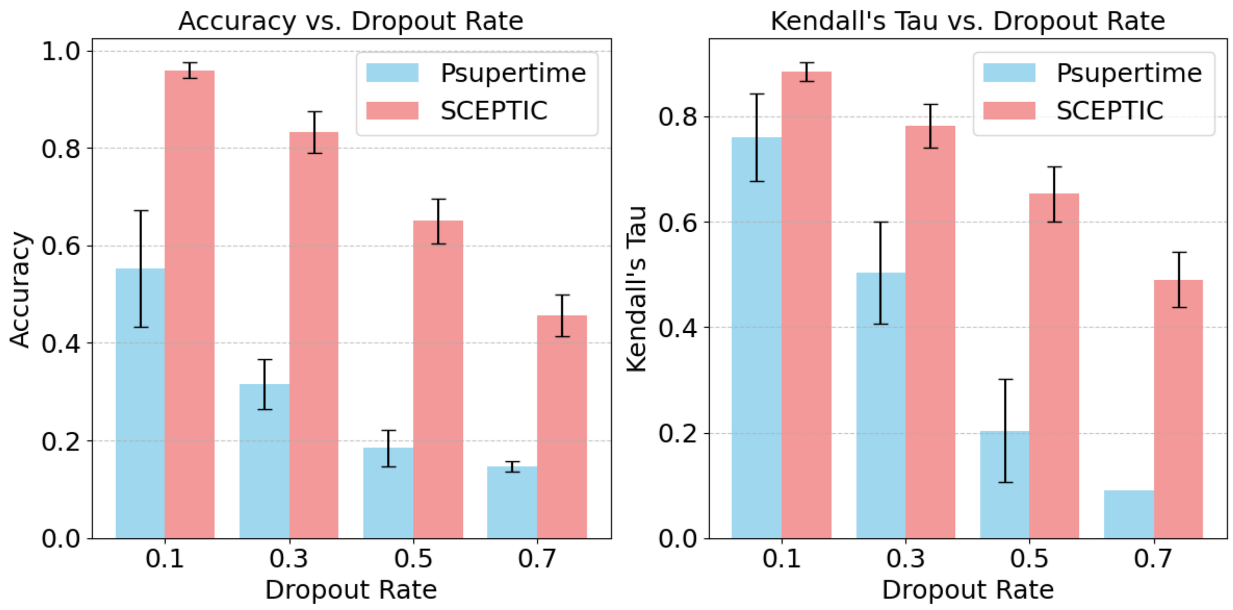

Figure S2: **Simulations with varying dropout rates.** We conducted additional scRNA-seq simulations incorporating varying dropout rates. Sceptic demonstrates greater robustness across different dropout rates compared to psupertime. The accuracy drop is significant in the presence of severe dropout conditions, suggesting room for improvement. One potential approach to mitigate this issue could involve applying imputation techniques, such as MAGIC to preprocess the data and reduce the impact of sparsity before applying Sceptic. This could enhance the model's ability to accurately capture pseudotime trajectories in highly sparse datasets.

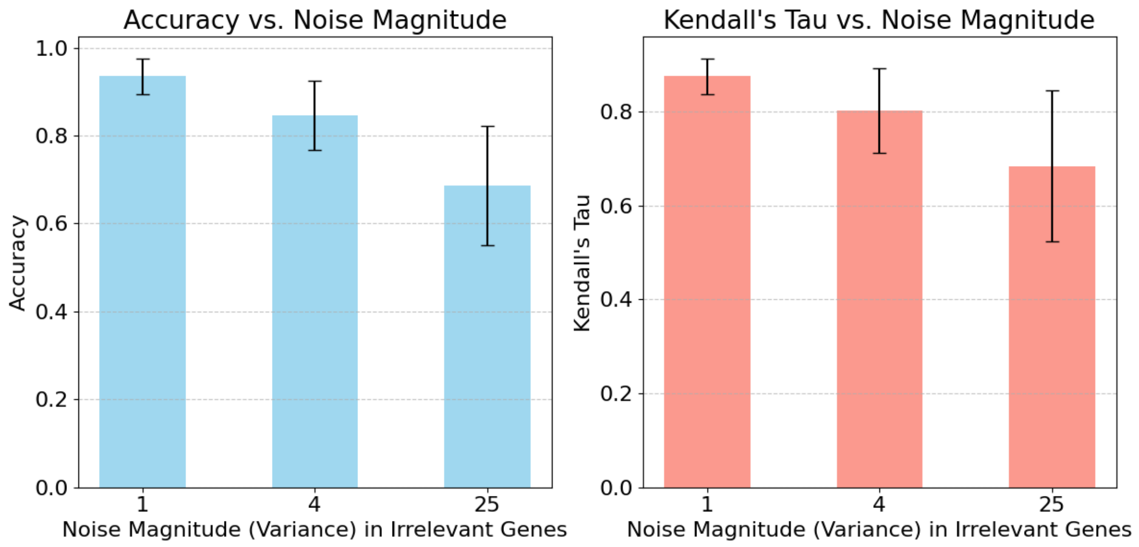

Figure S3: **Sceptic's performance under various noise magnitudes in irrelevant genes.** To evaluate Sceptic's robustness to irrelevant gene signals, we conducted additional experiments with varying noise magnitudes. We simulated irrelevant genes using Gaussian distributions with zero mean and increasing standard variance ($\sigma^2 \in \{1, 4, 25\}$). For each noise level, we generated 10 different datasets with random seeds and measured both accuracy and Kendall's τ. As expected, Sceptic's performance gradually decreases with increasing noise magnitude (Figure S3). These findings highlight both Sceptic's robustness and its limitations under different noise scenarios.

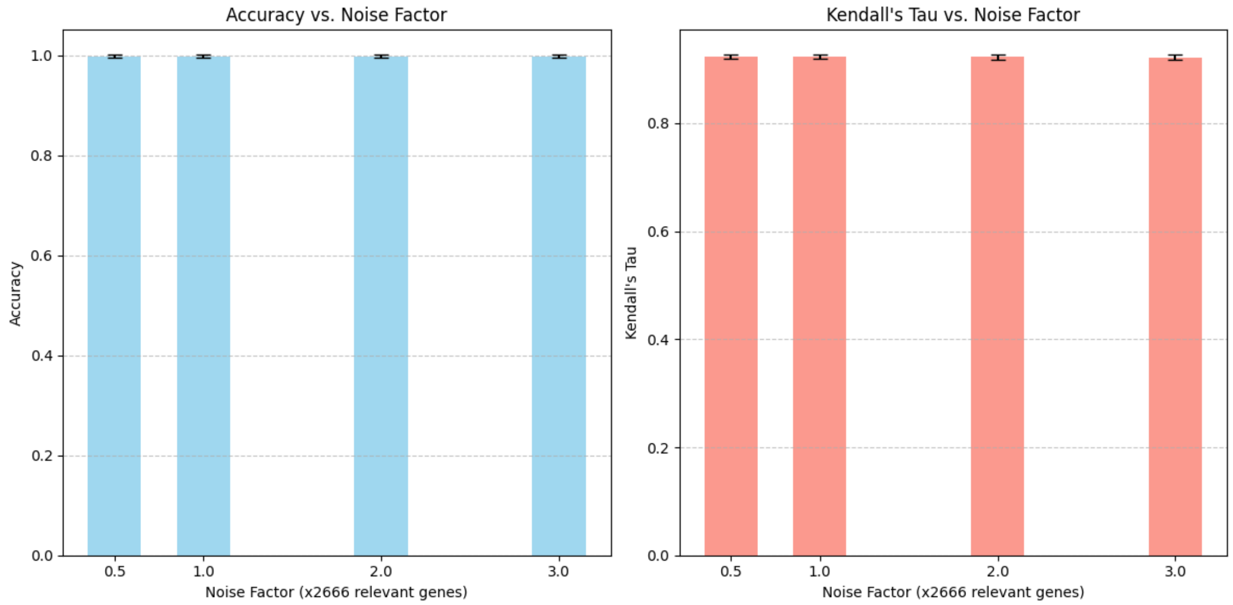

Figure S4: **Sceptic with varying numbers of irrelevant genes.** We conducted simulations by adding varying numbers of irrelevant genes to the dataset, following psupertime's simulation protocol. Specifically, we added varying numbers of irrelevant genes, corresponding to 0.5x, 1x, 2x, and 3x times the original 2,666 time-relevant genes. The results suggest that Sceptic is robust to irrelevant genes.

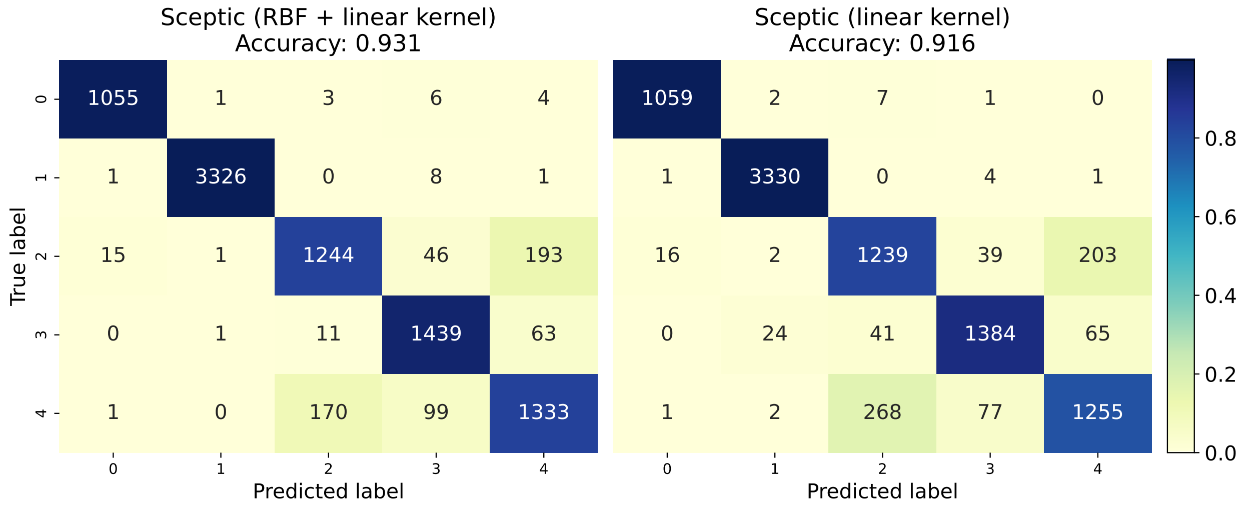

Figure S5: **Ablation study with and without non-linear kernels.** To test whether using a non-linear decision boundary improves classification performance, we evaluate two version of Sceptic on mESC data, with linear and non-linear (radial basis function) kernels.

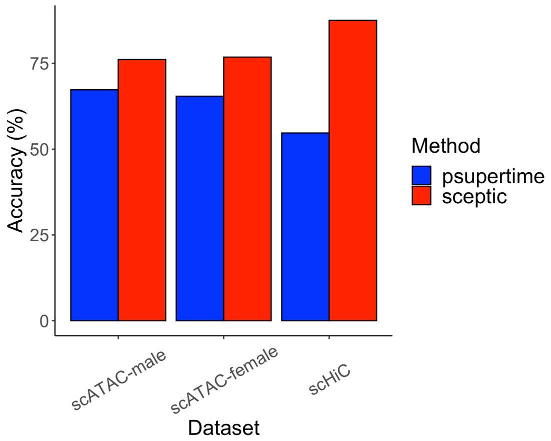

Figure S6: **Sceptic works well for scATAC-seq data.**

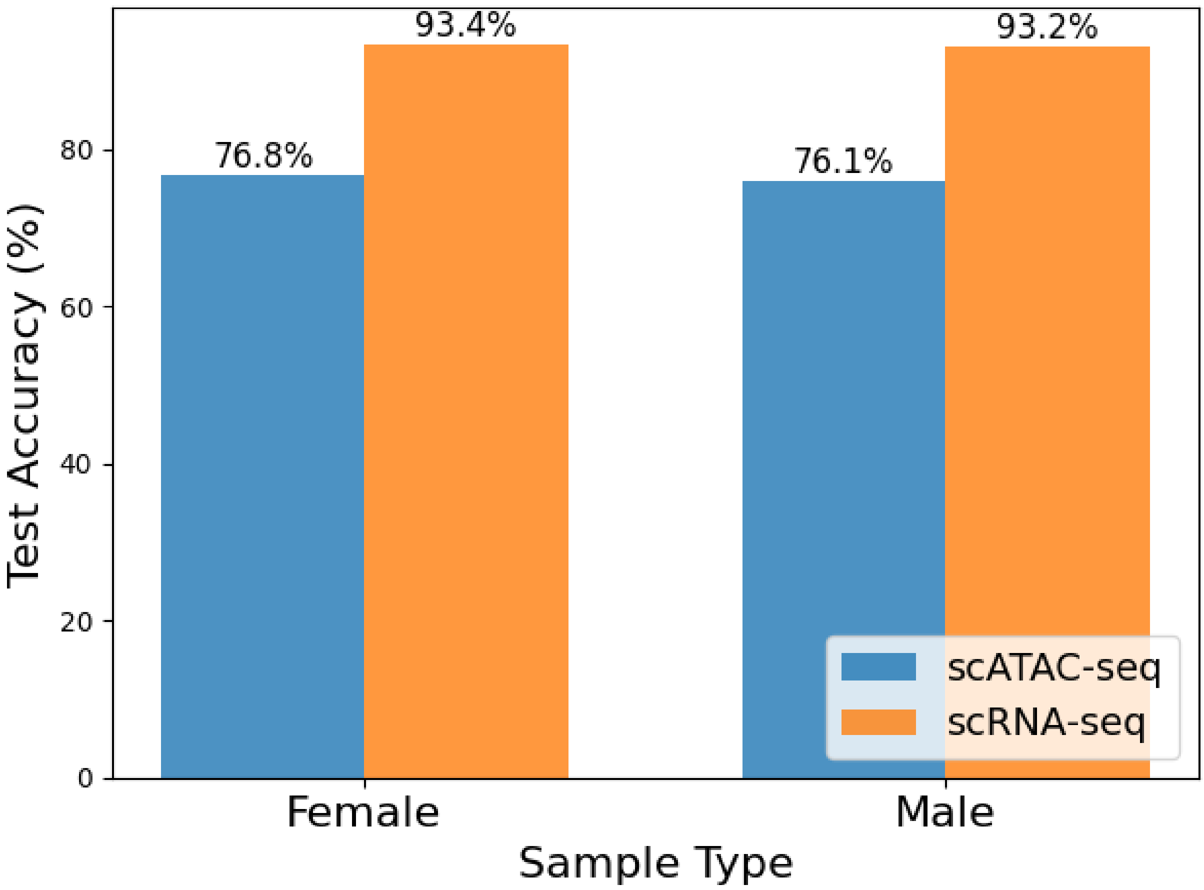

Figure S7: **Test accuracy across modalities.** The figure presents the cross-validated accuracy of Sceptic on scATAC-seq and scRNA-seq data from differentiating F121-6 (female) and F123 (male) mESCs.

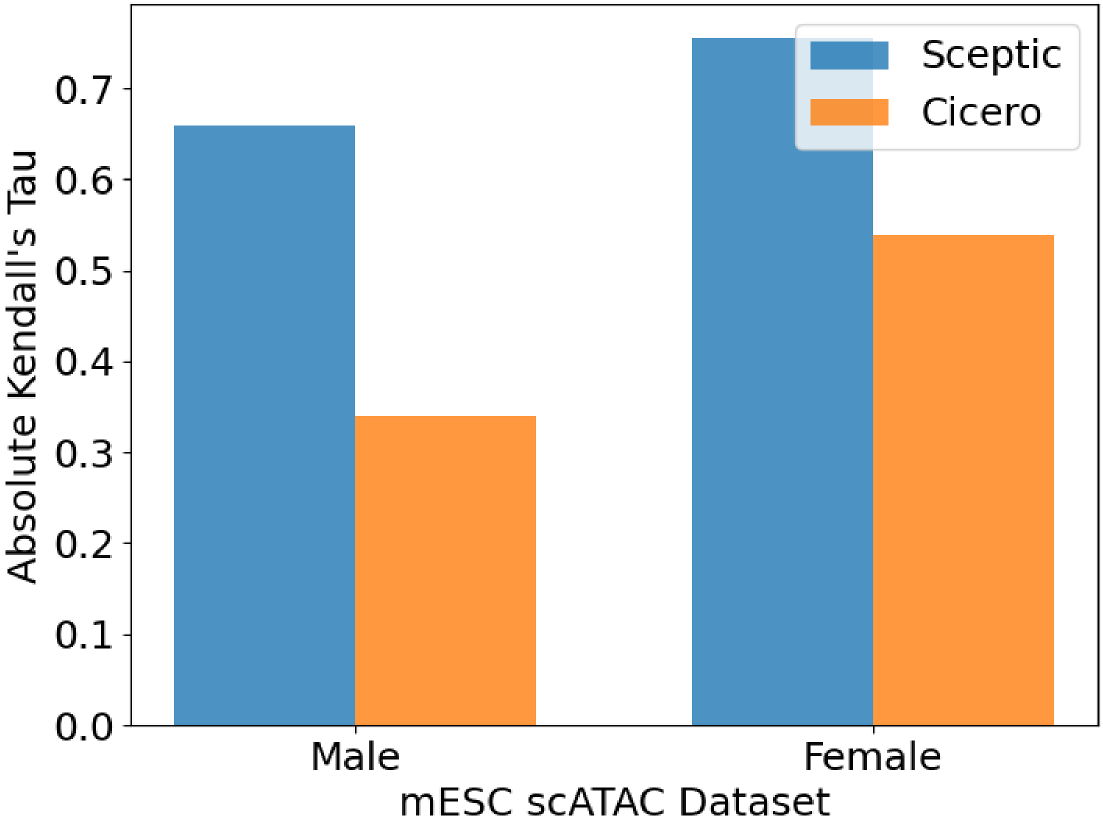

Figure S8: **Sceptic outperforms Cicero on mESC scATAC dataset.**

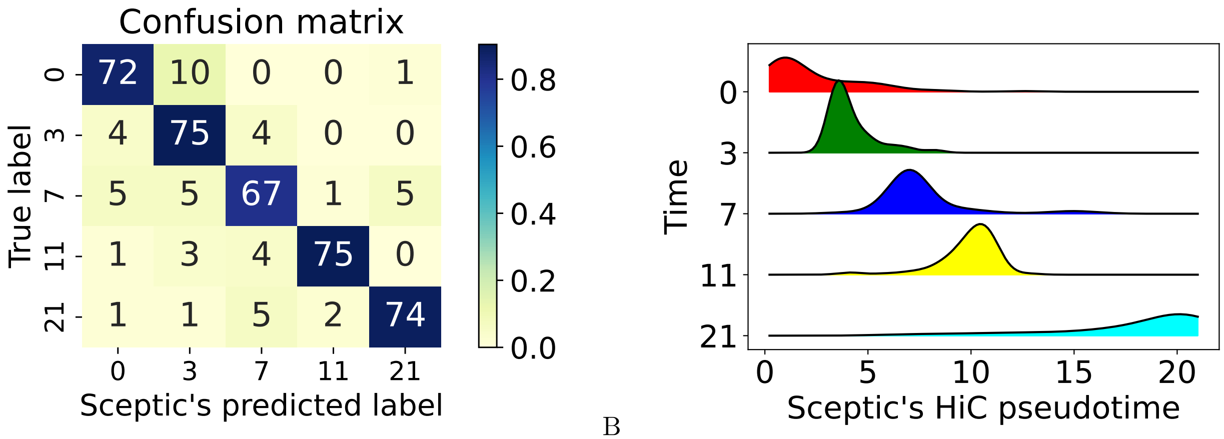

A B

Figure S9: **Sceptic works well for scHiC data.** We investigated whether Sceptic would generalize to single-cell HiC data. To do so, we collected allelic embryonic stem cell Tsixstop scHiC data from a recent study [1]. We downloaded the preprocessed contact decay profile for each chromosome and concatenated them across 20 chromosomes as the vector representation of each cell. To measure the classifier's performance, we randomly selected 83 cells from each time point, yielding 415 cells in total. Overall, Sceptic achieves 87.47% test set accuracy. **A** Sceptic can separate cells using allelic single-cell HiC data. **B** Ridge plot of Sceptic pseudotime, colored by the day of cells, on the Tsixstop data.

References

[1] G. Bonora, V. Ramani, R. Singh, H. Fang, D. Jackson, S. Srivatsan, R. Qiu, C. Lee, C. Trapnell, J. Shendure, et al. Single-cell landscape of nuclear configuration and gene expression during stem cell differentiation and x inactivation. *bioRxiv*, 2020.

[2] Will Macnair, Revant Gupta, and Manfred Claassen. psupertime: supervised pseudotime analysis for time-series single-cell RNA-seq data. *Bioinformatics*, 38(Supplement_1):i290–i298, 2022.
