# Supplementary material for: Sceptic: pseudotime analysis for time-series single-cell sequencing and imaging data
Source: Genome Biol. 2025 Jul 17;26:209. doi: 10.1186/s13059-025-03679-3 (PMC12273354; doi:10.1186/s13059-025-03679-3)
Supplement: Supplementary file 3 — Additional file 3. Review history. [file 13059_2025_3679_MOESM3_ESM.docx]

Review History

**First round of review**

**Reviewer 1**

**Were you able to assess all statistics in the manuscript, including the appropriateness of statistical tests used?**

Yes, and I have assessed the statistics in my report.

**Comments to author:**

In this paper, a new approach called Sceptic is presented which aims to infer "pseudotime" from time-series single cell RNA-seq data as well as other modalities such scATAC-seq, methylation, and also imaging data. While there are many approaches to infer pseudotime from a single scRNA-seq data relatively few exist when the data represent time points from a time-series experiment. Sceptic extends a previous method, which uses a supervised learning framework, psupertime, to incorporate a non-linear model and also use a different classification approach. Sceptic is based on an SVM one-vs-rest strategy, which is different from psupertime, which uses a single model. The overall approach is to treat the probability of a cell to have a particular time point stamp as the pseudotime value of a cell based on the assay-specific (scRNA-seq, scATAC-seq etc) measurements and define a loss that tries to minimize this. Sceptic is applied to simulated, scRNA-seq, scATAC-seq, methylation and also imaging data. Sceptic is compared to psupertime on simulated, selected scRNA-seq and scATAC-seq datasets and is shown to have a higher prediction accuracy. Sceptic is able to predict pseudotime from non-scRNA-seq data and in one application seemed to capture sex-specific differences. Overall, Sceptic is an interesting approach for predicting pseudotime while incorporating time series information. However, the experimental work is quite preliminary, there is limited comparison to other methods and the paper is lacking on providing novel insighsights from these results. More generally, the definition of pseudotime, which seems to be the probability of belonging to a particular timepoint could be better motivated. More details follow:

1. The authors compare only to psupertime and it is not clear how existing methods that ignore time and infer pseudotime for the entire dataset after concatenation perform and for that matter how does their definiiton of pseudotime related to what Sceptic defines pseudotime. To show the advantage of Sceptic additional experiments are needed to compare to a few well-known psuedotime estimation approaches, e.g., the original Monocle's pseudotime, diffusion pseudotime, PAGA or CellRank2's pseudotime.

2. The authors don't make any connection to other ways in which transitions in developmental systems are examined, e.g. with velocity, which has been extended to scATAC-seq too. The authors should try to better contextualize how their definition of pseudotime related to these other ways. This will also help motivate this approach better.

3. There is little to no effort for interpreting results. At a high level, it would be interesting to know why for example, a better time point classication is possible with scRNA-seq compared to scATAC-seq (Fig 5). It would also be helpful to understand how similar and different the predictions when considereing different modalities.

4. The methods are very sparse in how the dataset is processed for the classification model. This maybe in the supplemental methods, but the main methods need to provide more details.

5. The authors acknowledge that SVMs are not as interpretable as the linear models, but there are several tools for trying to interpret blackbox models to find what helps the prediction performance that could be explored ot examine the factors/genes that influence the predictions.

**Reviewer 2**

**Were you able to assess all statistics in the manuscript, including the appropriateness of statistical tests used?**

Yes: I have reviewed all statistical methods used in this paper

**Comments to author:**

The authors present Sceptic, a novel supervised machine learning approach for pseudotime analysis of time-series single-cell data, including scRNA-seq, scATAC-seq, and single-nucleus imaging. This method addresses limitations in existing tools such as psupertime, which relies on a linear model, by utilizing a support vector machine (SVM) framework. The use of SVM allows for non-linear decision boundaries and improved predictive accuracy. The manuscript is well-written, and the proposed method demonstrates significant performance improvements over current state-of-the-art tools. The approach has potential utility in the field; however, the authors should address the following points to strengthen the manuscript further:

1. Method Description:

The explanation of the method used to generate cell pseudotime requires additional clarity. For example, on page 3, line 60, it is stated that T_d is a time point, but how it is used as a weight in the formula is unclear. If E(Y|X=x) represents the probability of a cell belonging to a specific time point, further explanation is needed on how the model accounts for heterogeneity (pseudotime) within each time point.

2. Comparison with psupertime:

2.1 One of psupertime's strengths is its interpretability, as it provides ordering coefficients for genes to indicate their relevance to the label sequence. Does Sceptic offer a similar feature? Are the identified genes biologically meaningful?

2.2 Ablation studies are needed to validate the three key advantages of Sceptic over psupertime (page 6, line 33).

2.3 For Figure 3C, the accuracy values for psupertime appear to have been sourced from its original paper (Table 2 in psupertime), where the authors intentionally reduced the number of input genes to optimize runtime efficiency. For a fair comparison, consider using the Absolute Kendall's correlation coefficient as employed in the psupertime paper.

3. Simulation Experiments:

3.1 The simulation results are expected, as SVMs are generally well-suited for non-linear distributed data (e.g., bifurcating structures). To enhance the validity of the simulation, adopt more realistic strategies similar to those in psupertime (e.g., global time-series, cell-type time series, batch effects, and non-specific genes).

3.2 Simulations closer to real-world single-cell RNA-seq applications, including scenarios with multiple cell types and varying dropout rates, should be included for a more comprehensive evaluation.

3.3 In the simulation, it seems all 500 genes are relevant to time sequence, which is not reasonable in the real data. The authors should investigate the robustness of Sceptic to different numbers of irrelevant genes.

4. Real Data Experiments:

4.1 Most scRNA-seq datasets used in the study involve a single cell type, which does not reflect common real-world scenarios. The authors should evaluate Sceptic on more realistic datasets, such as the mouse gastrulation atlas (Nature 566, 490-495, 2019) or single-cell time-resolved mouse embryo data (Cell 184, 2825-2842.e22, 2021).

4.2 For non-RNA-seq data, the manuscript should include comparisons with competing methods to validate Sceptic's performance comprehensively.

5. Benchmarking with Competing Methods:

The study does not include comparisons with several state-of-the-art pseudotime inference tools that could handle timestamped scRNA-seq data. The authors should evaluate Sceptic against methods such as Mellon (Nat Methods, 2024), Tempora, embflow (Cell, 2021), MOSCOT (bioRxiv, 2023), and Waddington-OT (Cell, 2019).

Minor points

1. How Sceptic deal with the unbalanced number of cells from different time points? E.g. the cell number for a specific time point is much larger than the others.

2. While Sceptic performs well on intermediate-scale datasets, I suspect Sceptic could be applied to large datasets, due to the fact SVM is very slow to model large datasets. The experiment on time and RAM consumption should be included.

3. How many genes were used in the experiments on real data? Highly variable genes?

**Authors Response**

**Point-by-point responses to the reviewers’ comments:**

**Reviewer #1**

In this paper, a new approach called Sceptic is presented which aims to infer “pseudotime” from time-series single cell RNA-seq data as well as other modalities such scATAC-seq, methylation, and also imaging data. While there are many approaches to infer pseudotime from a single scRNA-seq data relatively few exist when the data represent time points from a time-series experiment. Sceptic extends a previous method, which uses a supervised learning framework, psupertime, to incorporate a non-linear model and also use a different classification approach. Sceptic is based on an SVM one-vs-rest strategy, which is different from psupertime, which uses a single model. The overall approach is to treat the probability of a cell to have a particular time point stamp as the pseudotime value of a cell based on the assay-specific (scRNA-seq, scATAC-seq etc) measurements and define a loss that tries to minimize this. Sceptic is applied to simulated, scRNA-seq, scATAC-seq, methylation and also imaging data. Sceptic is compared to psupertime on simulated, selected scRNA-seq and scATAC-seq datasets and is shown to have a higher prediction accuracy. Sceptic is able to predict pseudotime from non-scRNA-seq data and in one application seemed to capture sex-specific differences. Overall, Sceptic is an interesting approach for predicting pseudotime while incorporating time series information. However, the experimental work is quite preliminary, there is limited comparison to other methods and the paper is lacking on providing novel insighsights from these results. More generally, the definition of pseudotime, which seems to be the probability of belonging to a particular timepoint could be better motivated.

Thank you for this accurate summary of our contributions.

1. The authors compare only to psupertime and it is not clear how existing methods that ignore time and infer pseudotime for the entire dataset after concatenation perform and for that matter how does their definiiton of pseudotime related to what Sceptic defines pseudotime. To show the advantage of Sceptic additional experiments are needed to compare to a few well-known psuedotime estimation approaches, e.g., the original Monocle’s pseudotime, diffusion pseudotime, PAGA or CellRank2’s pseudotime.

You are correct that, in our submitted manuscript, we did not directly compare Sceptic to existing unsu­pervised methods. We made this decision because psupertime has previously been shown to perform as well or better than Monocle v2 on several datasets. However, in response to your comments above, we now include empirical comparisons with Monocle v2 and diffusion pseudotime (DPT). Furthermore, we note that CellRank2 relies on DPT’s pseudotime to infer its pseudotime kernel matrix. Therefore, we directly compared Sceptic with DPT-derived pseudotime and further demonstrated that Sceptic’s pseudotime can be seamlessly integrated with CellRank2 to infer transition matrices for downstream analysis.

Following is the text describing these new results, including the new panel Figure 10E:

We further evaluated Sceptic’s performance using Kendall’s *τ* coefficient, comparing with Psupertime and three state-of-the-art supervised methods, DPT [8], Palantir [23] and Monocle 2 [22]. In this analysis, we included two variants of Sceptic, one that includes the cross-validation procedure (Sceptic CV) and one without cross-validation (Sceptic noCV). In our added benchmarking results (Figure 10E), we found that the two supervised methods, Psupertime and Sceptic, consistently outperform all three unsupervised pseudotime inference methods. Furthermore, in the no cross-validation setting (which is comparable to Psupertime), Sceptic without cross-validation (Sceptic noCV) achieves a higher Kendall’s *τ* coefficient (Figure 10E). These results highlight the advantages of Sceptic’s pseudotime in leveraging supervised learning for pseudotime estimation.

2. The authors don’t make any connection to other ways in which transitions in developmental systems are examined, e.g. with velocity, which has been extended to scATAC-seq too. The authors should try to better contextualize how their definition of pseudotime related to these other ways. This will also help motivate

2

Figure 1: **Sceptic achieves better classification accuracy than Psupertime on single-cell RNA sequencing data. A** Confusion matrices for Sceptic (left) and psupertime (right) on the mESC data. Sceptic results use cross-validation, whereas psupertime results are from training on the full dataset. **B** Kernel density plot of Sceptic pseudotime, colored by the day of cells, on the mESC data. **C** Accuracy of both methods on six time-series datasets. Note that psupertime has previously been shown to perform comparably to or better than other methods—including Monocle 2, slingshot and Tempora—on five of these datasets. Thus, we only compare our performance with psupertime. **D** Confusion matrix from leave-one­time-point-out analysis. A successful model would tend to predict time points adjacent to the diagonal. **E** Comparison of performance using Kendal *τ* . Sceptic with cross-validation (Sceptic CV) outperforms or is comparable to most unsupervised methods. Sceptic without cross-validation (Sceptic noCV) achieves a higher Kendall *τ* coeffecient than psupertime. **F** Top 20 genes influencing prediction on mESC data, selected via mean absolute SHAP values.

3

this approach better.

In response to this request, we added the following text to the introduction of the manuscript:

An alternative means of characterizing cell state trajectories is via RNA velocity, which involves analyzing spliced and unspliced reads from scRNA-seq [1]. However, this approach requires full-length sequencing data with splicing information, which is unavailable in datasets from 3’-end sciRNA-seq or 5’-end 10x Genomics. Our method bypasses splicing, relying only on time-series labels to infer pseudotime trajectories. This makes Sceptic applicable across diverse platforms, offering a complementary approach to RNA velocity.

3. There is little to no effort for interpreting results. At a high level, it would be interesting to know why for example, a better time point classication is possible with scRNA-seq compared to scATAC-seq (Fig 5). It would also be helpful to understand how similar and different the predictions when considereing different modalities.

We agree that comparisons across modalities are important, and we appreciate the opportunity to clarify these results. As noted in the original manuscript, cells from scATAC-seq on days 7 and 11 exhibit similar chromatin accessibility patterns, making it challenging to distinguish these time points. In contrast, the corresponding scRNA-seq data reflects distinct gene expression patterns, which facilitates better classifica­tion. We have expanded this discussion in the revised manuscript to provide a more detailed comparison, as follows:

When comparing Sceptic’s performance across modalities, we found that Sceptic achieves better classification accuracy with scRNA-seq data compared to scATAC-seq data (Supplementary Figure 2). We speculate that this difference arises for two reasons. First, scRNA-seq directly captures transcriptomic states, which provide clear and biologically meaningful signals of cellular transitions. In contrast, chromatin accessibility data from scATAC-seq is sparser and thus noisier [26, 5], and is usually binarized to be closed or open, yielding a more limited dynamic range [3]. In many cases, an open chromatin region may remain open even when the genes it regulates are not expressed. For example, in embryonic stem cells, poised enhancers are associated with genes involved in lineage-specific differentiation. While they remain open and accessible but do not drive gene expression until the cell receives signals to differentiate into a specific cell type [28]. Second, scATAC-seq data is inherently sparser, with a significantly larger feature space compared to scRNA-seq (*>*200,000 peaks versus ∼20,000 genes). This increased sparsity and dimensionality make it more challenging for models to extract meaningful patterns for classification.

4. The methods are very sparse in how the dataset is processed for the classification model. This maybe in the supplemental methods, but the main methods need to provide more details.

In response to this request, we have moved and revised key details about the preprocessing of both sequencing data and imaging data from the supplemental methods into the main manuscript.

**Imaging data**

**Sample Collection** F121-6 mouse embryonic stem cells (ESCs) were grown and differentiated into em-bryoid bodies (EBs) as described in Bonora *et al.* [2]. Neural progenitor cells (NPCs) were derived from EBs at day 11 and collected at day 21. ESCs and EBs at days 3, 7, and 11, as well as NPCs were collected and frozen in 10% DMSO in 2–3 million cell aliquots.

4

Figure 2: **Test accuracy across modalities.** The figure presents the cross-validated accuracy of Sceptic on scATAC-seq and scRNA-seq data from differentiating F121-6 (female) and F123 (male) mESCs.

**Immunostaining** Frozen cell aliquots were thawed at 37 C and resuspended in 1x DPBS. Approximately 250,000 cells were plated on a 24-well glass bottom, black-walled plates (CellVis, Mountain View, CA, P24­1 .5H-N) via centrifugation at 300g for 5 mins. The cells were then fixed with 4% paraformaldehyde (Electron Microscopy Sciences Cat. No. 50-980-492) for 15 m at room temperature. After fixation, they were washed three times with 1x PBS and incubated in blocking buffer (3% BSA and 0.25% Tx100 in 1x PBS) for 1 hour at room temperature. They were then incubated with primary antibodies (Polyclonal nucleolin antibodies, 1:500, Invitrogen Cat. No.PA5-85972) diluted in blocking buffer for an hour at room temperature. The cells were washed three times with 1x PBS and incubated with secondary antibodies (Donkey anti rabbit Alexa 647, Invitrogen, Cat. No. A-31573) and DAPI diluted in blocking buffer for an hour at room temperature. They were then washed three times with 1x PBS and stored in 600 µL of 1x PBS.

**Image acquisition** Image acquisition was performed with an Automated Leica DMi8 inverted microscope with Adaptive Focus technology configured with a 40X 0.95 NA objective. The microscope was illuminated using a six-line Lumencor Spectra X Light Engine LED with Semrock multi-band dichroic filters (Spectra Services, Ontario, NY). The images were captured with a Zyla 4.2 sCMOS camera (Andor, Windsor, CT, USA) and the Piezo-driven stage (Okolab) was used to acquire z-stacks.

**Image segmentation** Nuclear segmentation was performed with a pre-trained Mask R-CNN model [4, 9] with a nuclear size cutoff of 900. Each nucleus was padded and cropped to a 128×128 pixel image.

**Image embeddings** For each single-nucleus image crop (128×128) of mESC dataset that we collected, we use pre-trained (on ImageNet [12]) Resnet34 [10](pytorch/vision:v0.10.0) from Pytorch [17] to generate a less-noisy vector-representation of the image. We generate 512-dim embeddings from the nucleolin-tagged

5

image and 512-dim embeddings from the DAPI mask image. By concatenating them together, we generated a 1024-dim representation for each image crop.

**scATAC-seq data**

We followed the epiScanpy pipeline to process the mESC scATAC-seq data [6]. Briefly, we first binarized the cell by peak matrix and identified the most variable peaks. The standard library size normalization and log normalization were performed. Finally, we used the top 50 principal components from normalized data as the input for our Sceptic model.

| **scRNA-seq data** |  |  |  |  |  |
| --- | --- | --- | --- | --- | --- |
| Datasets | Type | Time Label | # time points | # cells | Accession |
| mESC [2] | sciRNA | Embryonic day | 5 | 4064 | GSE184554 |
| hESC [18] | scRNA | Embryonic day | 5 | 1529 | E-MTAB-3929 |
| Human germline [13] | scRNA | Age (weeks) | 12 | 992 | GSE86146 |
| Embryonic beta cells [21] | scRNA | Developmental stage | 7 | 575 | GSE87375 |
| Acinar cells [7] | scRNA | Donor age | 8 | 411 | GSE81547 |
| MEF to neurons [25] | scRNA | Days since induction | 5 | 315 | GSE67310 |

Table 1: **Time-series scRNA-seq datasets.**

We used six publicly available scRNA-seq data sets (Table 1). We downloaded the five scRNA-seq datasets that were previously analyzed by Manair *et al.* from the R package “p supplementary” [(https://github. com/wmacnair/psupplementary)](https://github.com/wmacnair/psupplementary). We also downloaded the mESC scRNA-seq and scATAC-seq datasets an-alyzed by Bonora *et al.* [2] from [https://bitbucket.org/noblelab/mouse-sci-omics/src/master/.](https://We) We downloaded the processed scGEM dataset from UnionCom’s GitHub page [(https://github.com/caokai1073/ UnionCom/tree/master/scGEM)](https://github.com/caokai1073/UnionCom/tree/master/scGEM). For scRNA-seq data, we follow the standard preprocessing pipeline of Scanpy, which includes filtering low-quality cells, log-normalization and the identification of highly variable genes (default 1000 genes). Note that if fewer than 1000 genes are provided for a given data set, then all the genes are used for pseudotime inference.

5. The authors acknowledge that SVMs are not as interpretable as the linear models, but there are several tools for trying to interpret blackbox models to find what helps the prediction performance that could be explored ot examine the factors/genes that influence the predictions.

We agree that this is an interesting direction to pursue, and accordingly, we have now incorporated SHAP (SHapley Additive exPlanations) values to identify the important genes and factors that influence Sceptic’s predictions. Following is the text describing this new analysis, including the new panel Figure 10F:

To identify the genes that are important for Sceptic’s predictions, we calculated SHAP (SHapley Additive exPlanations) values [15] to evaluate feature importance (Figur 10F). By computing the mean absolute SHAP value across different categories, we identified the top 20 genes that contribute most to prediction performance. We analyzed the association of each of these genes with Gene Ontology annotations and the imprinting database (Supplementary Table 1) and identifed several trends. Notably, the genes *Airn*, *Meg3*, and *Kcnq1ot1* are well known imprinting genes [26], which suggests a connection to genomic imprinting processes during mouse embryonic differentiation; *Nrxn1* [11], *Npas3* [14], *Adgrl3* [27], and *Grip1* [24] are strongly associated with synaptic function and brain development; *Tet1* and *Meg3* are involved in epige­netic modifications during development. These findings not only provide biological insights into the genes influencing classification but also suggest potential links between genomic imprinting, neurodevelopmental processes, and epigenetic regulation during embryonic differentiation.

6

**Reviewer #2**

The authors present Sceptic, a novel supervised machine learning approach for pseudotime analysis of time-series single-cell data, including scRNA-seq, scATAC-seq, and single-nucleus imaging. This method addresses limitations in existing tools such as psupertime, which relies on a linear model, by utilizing a support vector machine (SVM) framework. The use of SVM allows for non-linear decision boundaries and improved predictive accuracy. The manuscript is well-written, and the proposed method demonstrates significant performance improvements over current state-of-the-art tools. The approach has potential utility in the field; however, the authors should address the following points to strengthen the manuscript further

Thank you for your thoughtful and constructive feedback. We have carefully addressed the points you raised, as outlined below.

1. Method Description: The explanation of the method used to generate cell pseudotime requires additional clarity. For example, on page 3, line 60, it is stated that *Td* is a time point, but how it is used as a weight in the formula is unclear. If *E*(*Y |X* = *x*) represents the probability of a cell belonging to a specific time point, further explanation is needed on how the model accounts for heterogeneity (pseudotime) within each time point.

We apologize for the lack of clarity in the original manuscript. We have revised the text to make the explanation more precise. *Td* is indeed a time point, and it is used as a weight in the formula for calculating the conditional expectation. We have revised the manuscript to make this clear.

We used SVM models to first estimate the probability of a cell belonging to each discrete time point *Td* given its observed gene expression profile. These probabilities are then used to compute the conditional expectation, which reflects the cell’s pseudotime as a weighted sum of the time points.

The probabilities *P* (*Y |X* = *x*) are defined as follows:

⠀

⠀⠀⠀

*p*1	if cell is collected from *T*1*,*

*p*2	if cell is collected from *T*2*,*

LI	.

**.**

LuLl

*pD* if cell is collected from *TD.*

*P* (*Y |X* = *x*) =

Using these probabilities, the pseudotime is calculated as:

*D*_P_	*D*_P_

*E*(*Y |X* = *x*) =	*Prob*(cell is collected from *Td|X* = *x*) *× Td* =	*pd × Td.*

*d=1	d=1*

This framework ensures that the model accounts for heterogeneity within each time point by assigning a pseudotime to each cell based on the conditional expectation of its time point assignment.

2. Comparison with psupertime: 2.1 One of psupertime’s strengths is its interpretability, as it provides ordering coefficients for genes to indicate their relevance to the label sequence. Does Sceptic offer a similar feature? Are the identified genes biologically meaningful?

Although Sceptic does not provide gene ordering coefficients directly, in the revised manuscript we have leveraged SHAP values, which are widely used to interpret machine learning models, to help make the results more interpretable. SHAP values allow us to identify the most influential features (genes) contributing to the classification performance, thereby offering a biologically meaningful interpretation of our model’s

7

predictions. We have incorporated this change into our revised manuscript, along with the new panel Figure 10F.

To identify the genes that are important for Sceptic’s predictions, we calculated SHAP (SHapley Additive exPlanations) values [15] to evaluate feature importance (Figur 10F). By computing the mean absolute SHAP value across different categories, we identified the top 20 genes that contribute most to prediction performance. We analyzed the association of each of these genes with Gene Ontology annotations and the imprinting database (Supplementary Table 1) and identifed several trends. Notably, the genes *Airn*, *Meg3*, and *Kcnq1ot1* are well known imprinting genes [26], which suggests a connection to genomic imprinting processes during mouse embryonic differentiation; *Nrxn1* [11], *Npas3* [14], *Adgrl3* [27], and *Grip1* [24] are strongly associated with synaptic function and brain development; *Tet1* and *Meg3* are involved in epige­netic modifications during development. These findings not only provide biological insights into the genes influencing classification but also suggest potential links between genomic imprinting, neurodevelopmental processes, and epigenetic regulation during embryonic differentiation.

2.2 Ablation studies are needed to validate the three key advantages of Sceptic over psupertime (page 6, line 33).

We agree with this suggestion, and we carried two of the three suggested ablation studies. However, the third feature that we believe contributes to Sceptic’s performance is its use of multiple score functions, which inherently increases its capacity to model complex, non-linear data compared to psupertime’s single-threshold linear model. While we considered various ways to isolate this component for ablation, we were unable to design a clean ablation experiment to directly quantify its effect. The other two ablations are described as follows:

To further assess Sceptic’s performance, we carried out two ablation experiments. First, to assess the impact of allowing non-linear decision boundaries, we implemented a version of Sceptic using an SVM model with only linear kernels. This version showed a performance drop of 1.5% in terms of both accuracy compared to the full version of Sceptic (Supplementary Figure 3). Second, we evaluated the role of nested cross-validation by comparing the performance of Sceptic with and without this feature. As shown in Figure 10E, the version without cross-validation consistently underperforms the version with nested cross-validation, as expected. Thus, by incorporating nested cross-validation, Sceptic prevents overfitting on the training data and achieves more reliable performance on unseen data.

2.3 For Figure 3C, the accuracy values for psupertime appear to have been sourced from its original paper (Table 2 in psupertime), where the authors intentionally reduced the number of input genes to optimize runtime efficiency. For a fair comparison, consider using the Absolute Kendall’s correlation coefficient as employed in the psupertime paper.

You are correct that those values came from the original psupertime paper. As you suggested, we have added the absolute Kendall’s correlation coefficient to our analysis, as employed in the original psupertime paper. We have updated the manuscript to reflect this comparison:

We further evaluated Sceptic’s performance using Kendall’s *τ* coefficient, comparing with Psupertime and three state-of-the-art supervised methods, DPT [8], Palantir [23] and Monocle 2 [22]. In this analysis, we included two variants of Sceptic, one that includes the cross-validation procedure (Sceptic CV) and one without cross-validation (Sceptic noCV). In our added benchmarking results (Figure 10E), we found that the two supervised methods, Psupertime and Sceptic, consistently outperform all three unsupervised pseudotime inference methods. Furthermore, in the no cross-validation setting (which is comparable to Psupertime), Sceptic without cross-validation (Sceptic noCV) achieves a higher Kendall’s *τ* coefficient (Figure 10E). These results highlight the advantages of Sceptic’s pseudotime in leveraging supervised learning for pseudotime

8

Figure 3: **Ablation study with and without non-linear kernels.** To test whether using a non-linear decision boundary improves classification performance, we evaluate two version of Sceptic on mESC data, with linear and non-linear (radial basis function) kernels.

estimation.

3. Simulation Experiments: 3.1 The simulation results are expected, as SVMs are generally well-suited for non-linear distributed data (e.g., bifurcating structures). To enhance the validity of the simulation, adopt more realistic strategies similar to those in psupertime (e.g., global time-series, cell-type time series, batch effects, and non-specific genes).

As suggested, we have updated the manuscript to include enhanced simulations. These simulations are referenced in the main text as follows:

To further enhance the simulation, we adopt the framework in psupertime, where we incorporated global time series, cell type time series, batch effects, and non-specific genes (see the descriptions in Section 2.6 of [16]). Our results show that Sceptic outperforms or is comparable to psupertime across 20 different randomly generated datasets (Supplementary Figure 5). Moreover, to evaluate Sceptic’s robustness in more realistic single-cell RNA-seq scenarios, we conducted additional simulations incorporating varying dropout rates (Supplementary Figure 6). Finally, we evaluated Sceptic’s time and RAM consumption for datasets of varying sizes (Supplementary Figure 7).

Here are the corresponding changes in the supplement:

**Sceptic outperforms psupertime using psupertime’s simulation frame­work.**

To further expand our simulations, we adopted strategies inspired by those used by psupertime. Specifically, we incorporated global time-series trajectories, cell-type-specific time series, batch effects, and the inclusion of non-specific genes into simulation framework, as described by [16]. However, whereas the original psupertime simulation used one of the real datasets as a reference to compute the summary statistics that underlie the

9

Figure 4: **Sceptic achieves better classification accuracy than Psupertime on single-cell RNA sequencing data. A** Confusion matrices for Sceptic (left) and psupertime (right) on the mESC data. Sceptic results use cross-validation, whereas psupertime results are from training on the full dataset. **B** Kernel density plot of Sceptic pseudotime, colored by the day of cells, on the mESC data. **C** Accuracy of both methods on six time-series datasets. Note that psupertime has previously been shown to perform comparably to or better than other methods—including Monocle 2, slingshot and Tempora—on five of these datasets. Thus, we only compare our performance with psupertime. **D** Confusion matrix from leave-one­time-point-out analysis. A successful model would tend to predict time points adjacent to the diagonal. **E** Comparison of performance using Kendal *τ* . Sceptic with cross-validation (Sceptic CV) outperforms or is comparable to most unsupervised methods. Sceptic without cross-validation (Sceptic noCV) achieves a higher Kendall *τ* coeffecient than psupertime. **F** Top 20 genes influencing prediction on mESC data, selected via mean absolute SHAP values.

10

Figure 5: **Comparison of Sceptic and psupertime using the psupertime simulation framework.** Each dot represents a simulated dataset generated with a different random seed. We evaluated Sceptic against psupertime based on accuracy and Kendall’s tau coefficient. Dots positioned below the diagonal indicate superior performance by Sceptic. A Wilcoxon rank test was used to statistically compare the models across 20 randomly simulated datasets.

simulation, for simplicity we randomly sample two summary statistics, log means and dispersion from a Gaussian distribution (with mean = 1, variance = 0.5) and Gamma distribution (with shape = 2, scale = 0.5), respectively. Under these simulation scenarios, Sceptic consistently demonstrated robust performance, outperforming psupertime for all 20 random seeds across multiple metrics, including classification accuracy and Kendall’s *τ* coefficient (Figure 5). These results further validate Sceptic’s ability to handle complex, real-world data.

3.2 Simulations closer to real-world single-cell RNA-seq applications, including scenarios with multiple cell types and varying dropout rates, should be included for a more comprehensive evaluation.

We have included these new simulations and their results in Section 3.1 of the revised manuscript and provided further details in the corresponding Figure 6.

3.3 In the simulation, it seems all 500 genes are relevant to time sequence, which is not reasonable in the real data. The authors should investigate the robustness of Sceptic to different numbers of irrelevant genes.

We have made the requested changes to the simulation, and we have updated Supplementary Figure 7 to include these new results.

4. Real Data Experiments: 4.1 Most scRNA-seq datasets used in the study involve a single cell type, which does not reflect common real-world scenarios. The authors should evaluate Sceptic on more realistic datasets, such as the mouse gastrulation atlas (Nature 566, 490-495, 2019) or single-cell time-resolved mouse embryo data (Cell 184, 2825-2842.e22, 2021).

11

Figure 6: **Simulations with varying dropout rates.** We conducted additional scRNA-seq simulations incorporating varying dropout rates. Sceptic demonstrates greater robustness across different dropout rates compared to psupertime. The accuracy drop is significant in the presence of severe dropout conditions, suggesting room for improvement. One potential approach to mitigate this issue could involve applying imputation techniques, such as MAGIC to preprocess the data and reduce the impact of sparsity before applying Sceptic. This could enhance the model’s ability to accurately capture pseudotime trajectories in highly sparse datasets.

Figure 7: **Sceptic with varying numbers of irrelevant genes.** We conducted simulations by adding varying numbers of irrelevant genes to the dataset, following psupertime’s simulation protocol. Specifically, we added varying numbers of irrelevant genes, corresponding to 0.5x, 1x, 2x, and 3x times the original 2,666 time-relevant genes. The results suggest that Sceptic is robust to irrelevant genes.

12

To clarify, several datasets used in our study include multiple cell types. For example, the mESC dataset includes three cell types: mouse embrynoic stem cells, nuclear bodies, and neural progentitor cells. The MEF dataset includes MEF, myocyte, and fibroblast cell types. We acknowledge that the scalability of our current implementation limits Sceptic to small or intermediate-sized datasets (*<* 10*,* 000 cells). Applying Sceptic to large atlas datasets, such as the mouse gastrulation atlas or single-cell time-resolved mouse embryo data, would require downsampling or improvements in computational efficiency. For example, Dai *et al.* recently developed ReHLine, which scales to 10^6^ samples, and integrating such approaches could be explored in future work. In this study, to address your suggestion, we performed an analysis using a downsampled version of the mouse embryogenesis atlas (Qiu et al., Nature 2024):

**Applying Sceptic to mouse atlas data**

We performed pseudotime analysis via Sceptic using a downsampled version of the mouse embryogenesis atlas [20] To do so, we randomly selected 200 cells from each of the 42 embryonic stages and used the resulting data for pseudotime inference. We found that Sceptic accurately predicts most cells close to the diagonal, which reflects the temporal proximity of neighboring time points (Figure 8A). This result highlights Sceptic’s ability to preserve temporal structure, even when time labels are closely spaced. We also generated a UMAP embedding colored by Sceptic’s inferred pseudotime, which clearly demonstrates the temporal progression of cells along the pseudotime trajectory (Figure 8B–C). These results demonstrate Sceptic’s ability to handle multi-cell-type data and its potential to generalize to larger and more complex datasets.

4.2 For non-RNA-seq data, the manuscript should include comparisons with competing methods to validate Sceptic’s performance comprehensively.

As suggested, we have conducted additional analyses to compare Sceptic’s performance against existing methods for non-RNA-seq data where applicable.

- Imaging: We did not find pseudotime methods for imaging data, and thus we compared Sceptic with psupertime for imaging data. We demonstrated that Sceptic outperforms psupertime in terms of classification accuracy and pseudotime inference. This comparison highlights Sceptic’s versatility across modalities.
- scATAC-seq: For scATAC-seq, we compared Sceptic with Cicero, which uses a Monocle-like framework to infer pseudotime and found that Sceptic consistently outperformed Cicero. These results are included in Section 3.4, along with a discussion of the challenges posed by Cicero’s implementation:

For scATAC-seq analysis, we compared Sceptic with Cicero—a Monocle-based framework [19] for pseudotime inference—using both male and female mESC data. We found that Cicero requires manual selection of the root cell and often assigns infinite pseudotime values to cells positioned far from the starting point in the trajectory, reducing its robustness for this type of analysis. As shown in Supplementary Fig. 9, our Sceptic model consistently outperforms Cicero, achieving a higher mean absolute Kendall’s Tau coefficient by 0.22 to 0.31 for both male and female datasets.

- scHi-C: We are aware of scHiCPTR, a pseudotime inference method designed for analyzing cell cycle dynamics using scHi-C data. However, scHiCPTR focuses on a specific task (inferring pseudotime within the context of cell cycle dynamics) rather than global temporal inference across a broader trajectory. This distinction makes it less applicable to the scope of our work, which emphasizes global temporal relationships across diverse modalities. Nonetheless, Sceptic’s ability to generalize to scHi-C data demonstrates its flexibility for broader applications.
- Other modalities (e.g., scMethylation): We did not identify established pseudotime methods specifically designed for scMethylation data. This highlights the need for further development of tools applicable

Figure 8: **Sceptic applied to an atlas of mouse embryogenesis. A** Confusion matrix for Sceptic on the mouse embryogenesis atlas data. **B** UMAP of cells of the mouse embryogenesis atlas data, colored by cell type. **C** UMAP of cells of the mouse embryogenesis atlas data, colored by Sceptic’s inferred pseudotime.

| A |  |
| --- | --- |

Figure 9: **Sceptic outperforms Cicero on mESC scATAC dataset.**

to such modalities. Sceptic’s ability to analyze such data underscores its adaptability and potential utility in new contexts.

5. Benchmarking with Competing Methods: The study does not include comparisons with several state-of-the-art pseudotime inference tools that could handle timestamped scRNA-seq data. The authors should evaluate Sceptic against methods such as Mellon (Nat Methods, 2024), Tempora, embflow (Cell, 2021), MOSCOT (bioRxiv, 2023), and Waddington-OT (Cell, 2019).

To address this, we have included comparisons with other methods where possible. Tempora and Monocle2 were previously compared to psupertime in the psupertime paper. In our revised manuscript, we adopted this evaluation and included the reported performance metrics in our analysis. To evaluate Sceptic against Mellon, we directly compared it to Palantir, because Palantir forms the core of Mellon’s pseudotime computation. We also compared with DPT (Diffusion Pseudotime), which is a what CellRank2 uses pseudotime inference. While we acknowledge the relevance of additional tools such as embflow (Cell, 2021), MOSCOT (Nature, 2025), and Waddington-OT (Cell, 2019), we did not have sufficient time or resources to evaluate Sceptic against these methods in this study. We will consider including these comparisons in future work as part of a broader benchmarking effort. We have updated the manuscript to include the new comparisons and have incorporated the corresponding results in Figure 10.

We further evaluated Sceptic’s performance using Kendall’s *τ* coefficient, comparing with Psupertime and three state-of-the-art supervised methods, DPT [8], Palantir [23] and Monocle 2 [22]. In this analysis, we included two variants of Sceptic, one that includes the cross-validation procedure (Sceptic CV) and one without cross-validation (Sceptic noCV). In our added benchmarking results (Figure 10E), we found that the two supervised methods, Psupertime and Sceptic, consistently outperform all three unsupervised pseudotime inference methods. Furthermore, in the no cross-validation setting (which is comparable to Psupertime), Sceptic without cross-validation (Sceptic noCV) achieves a higher Kendall’s *τ* coefficient (Figure 10E). These results highlight the advantages of Sceptic’s pseudotime in leveraging supervised learning for pseudotime estimation.

Minor points 1. How Sceptic deal with the unbalanced number of cells from different time points? E.g. the cell number for a specific time point is much larger than the others.

To address the issue of imbalance in the number of cells across time points, we apply downsampling to ensure an equal number of cells per time point. We have revised the manuscript to make this clearer.

Figure 10: **Sceptic achieves better classification accuracy than Psupertime on single-cell RNA sequencing data. A** Confusion matrices for Sceptic (left) and psupertime (right) on the mESC data. Sceptic results use cross-validation, whereas psupertime results are from training on the full dataset. **B** Kernel density plot of Sceptic pseudotime, colored by the day of cells, on the mESC data. **C** Accuracy of both methods on six time-series datasets. Note that psupertime has previously been shown to perform comparably to or better than other methods—including Monocle 2, slingshot and Tempora—on five of these datasets. Thus, we only compare our performance with psupertime. **D** Confusion matrix from leave-one­time-point-out analysis. A successful model would tend to predict time points adjacent to the diagonal. **E** Comparison of performance using Kendal *τ* . Sceptic with cross-validation (Sceptic CV) outperforms or is comparable to most unsupervised methods. Sceptic without cross-validation (Sceptic noCV) achieves a higher Kendall *τ* coeffecient than psupertime. **F** Top 20 genes influencing prediction on mESC data, selected via mean absolute SHAP values.

Figure 11: **Time and RAM consumption of Sceptic.**

To address the issue of imbalance in the number of cells across time points, we randomly downsampled the number of cells from each time point to the smallest cell number across all time points, which left us with 535 female cells (107 cells for each time point) and 560 male cells (140 cells for each time point).

2. While Sceptic performs well on intermediate-scale datasets, I suspect Sceptic could be applied to large datasets, due to the fact SVM is very slow to model large datasets. The experiment on time and RAM consumption should be included.

Indeed, the scalability of SVMs can present challenges for large datasets. Our current implementation of Sceptic is optimized for intermediate-scale datasets (*<* 10*,* 000 cells). Scaling Sceptic to handle datasets with 10^6^ cells or more would require significant computational optimizations, such as utilizing approximate SVM implementations or distributed computing frameworks. We plan to address these challenges in future work. In the meantime, we have included an evaluation of time and RAM consumption for datasets of varying sizes (ranging from 1,000 to 4,000 cells) in the revised manuscript.

We also evaluated Sceptic’s time and RAM consumption for datasets of varying sizes, ranging from 1000 to 4000 cells. These results provide insights into Sceptic’s computational efficiency on datasets of practical sizes. The results (Figure reffigr1:RAM) show that Sceptic’s resource requirements increase linearly with the number of cells. This analysis highlights the strengths and current limitations of Sceptic while providing a foundation for future work aimed at improving scalability.

3. How many genes were used in the experiments on real data? Highly variable genes?

For most datasets, we used the top 1000 highly variable genes. This approach ensures that the analysis focuses on the most informative features while reducing noise from less variable genes. For datasets with fewer than 1000 genes (e.g., scGEM), we used all available genes. This choice was made to ensure that the model could leverage all the information present in such datasets. We have clarified these points in the revised manuscript:

For scRNA-seq data, we follow the standard preprocessing pipeline of Scanpy, which includes filtering low-quality cells, log-normalization and the identification of highly variable genes (default 1000 genes). Note that if fewer than 1000 genes are provided for a given data set, then all the genes are used for pseudotime inference.

17

**References**

[1] Volker Bergen, Marius Lange, Stefan Peidli, F Alexander Wolf, and Fabian J Theis. Generalizing rna velocity to transient cell states through dynamical modeling. *Nature biotechnology*, 38(12):1408–1414, 2020.
[2] G. Bonora, V. Ramani, R. Singh, H. Fang, D. Jackson, S. Srivatsan, R. Qiu, C. Lee, C. Trapnell, J. Shendure, et al. Single-cell landscape of nuclear configuration and gene expression during stem cell differentiation and x inactivation. *bioRxiv*, 2020.
[3] Jason D Buenrostro, Beijing Wu, Ulrike M Litzenburger, Dave Ruff, Michael L Gonzales, Michael P Snyder, Howard Y Chang, and William J Greenleaf. Single-cell chromatin accessibility reveals principles of regulatory variation. *Nature*, 523(7561):486–490, 2015.
[4] Juan C Caicedo, Allen Goodman, Kyle W Karhohs, Beth A Cimini, Jeanelle Ackerman, Marzieh Haghighi, CherKeng Heng, Tim Becker, Minh Doan, Claire McQuin, et al. Nucleus segmentation across imaging experiments: the 2018 Data Science Bowl. *Nature Methods*, 16(12):1247–1253, 2019.
[5] Huidong Chen, Caleb Lareau, Tommaso Andreani, Michael E Vinyard, Sara P Garcia, Kendell Clement, Miguel A Andrade-Navarro, Jason D Buenrostro, and Luca Pinello. Assessment of computational methods for the analysis of single-cell atac-seq data. *Genome biology*, 20:1–25, 2019.
[6] Anna Danese, Maria L Richter, Kridsadakorn Chaichoompu, David S Fischer, Fabian J Theis, and Maria Colom´e-Tatch´e. Episcanpy: integrated single-cell epigenomic analysis. *Nature Communications*, 12(1):5228, 2021.
[7] Martin Enge, H Efsun Arda, Marco Mignardi, John Beausang, Rita Bottino, Seung K Kim, and Stephen R Quake. Single-cell analysis of human pancreas reveals transcriptional signatures of aging and somatic mutation patterns. *Cell*, 171(2):321–330, 2017.
[8] Laleh Haghverdi, Maren Bu¨ttner, F Alexander Wolf, Florian Buettner, and Fabian J Theis. Diffusion pseudotime robustly reconstructs lineage branching. *Nature methods*, 13(10):845–848, 2016.
[9] Kaiming He, Georgia Gkioxari, Piotr Dolla´r, and Ross Girshick. Mask r-cnn. In *Proceedings of the IEEE international conference on computer vision*, pages 2961–2969, 2017.
[10] Kaiming He, Xiangyu Zhang, Shaoqing Ren, and Jian Sun. Deep residual learning for image recognition. In *Proceedings of the IEEE conference on computer vision and pattern recognition*, pages 770–778, 2016.
[11] Zhonghua Hu, Xiao Xiao, Zhuohua Zhang, and Ming Li. Genetic insights and neurobiological implica­tions from nrxn1 in neuropsychiatric disorders. *Molecular psychiatry*, 24(10):1400–1414, 2019.
[12] Alex Krizhevsky, Ilya Sutskever, and Geoffrey E Hinton. Imagenet classification with deep convolutional neural networks. In *Advances in neural information processing systems*, pages 1097–1105, 2012.
[13] Li Li, Ji Dong, Liying Yan, Jun Yong, Xixi Liu, Yuqiong Hu, Xiaoying Fan, Xinglong Wu, Hongshan Guo, Xiaoye Wang, et al. Single-cell rna-seq analysis maps development of human germline cells and gonadal niche interactions. *Cell stem cell*, 20(6):858–873, 2017.
[14] Ji-Wei Liu, Han Li, and Yang Zhang. Npas3 regulates stemness maintenance of radial glial cells and neu­ronal migration in the developing mouse cerebral cortex. *Frontiers in Cellular Neuroscience*, 16:865681, 2022.
[15] S. M. Lundberg and S. Lee. A unified approach to interpreting model predictions. *Advances in Neural Information Processing Systems*, 2017.
[16] Will Macnair, Revant Gupta, and Manfred Claassen. psupertime: supervised pseudotime analysis for time-series single-cell RNA-seq data. *Bioinformatics*, 38(Supplement 1):i290–i298, 2022.

18

[17] A. Paszke, S. Gross, F. Massa, A. Lerer, J. Bradbury, G. Chanan, T. Killeen, Z. Lin, N. Gimelshein, L. Antiga, A. Desmaison, A. Kopf, E. Yang, Z. DeVito, M. Raison, A. Tejani, S. Chilamkurthy, B. Steiner, L. Fang, J. Bai, and S. Chintala. Pytorch: An imperative style, high-performance deep learning library. In *Advances in Neural Information Processing Systems 32*, pages 8024–8035. Curran Associates, Inc., Vancouver, Canada, 2019.

[18] S Petropoulos, SP Panula, JP Schell, and F Lanner. Single-cell rna sequencing: revealing human pre-implantation development, pluripotency and germline development. *Journal of internal medicine*, 280(3):252–264, 2016.
[19] H. A. Pliner, J. S. Packer, J. L. McFaline-Figueroa, D. A. Cusanovich, R. M. Daza, D. Aghamirzaie, S. Srivatsan, X. Qiu, D. Jackson, A. Minkina, A. C. Adey, F. J. Steemers, J. Shendure, and C. Trapnell. Cicero predicts cis-regulatory DNA interactions from single-cell chromatin accessibility data. *Molecular Cell*, 71(5):858–871, 2018.
[20] Chengxiang Qiu, Beth K Martin, Ian C Welsh, Riza M Daza, Truc-Mai Le, Xingfan Huang, Eva K Nichols, Megan L Taylor, Olivia Fulton, Diana R O’Day, et al. A single-cell time-lapse of mouse prenatal development from gastrula to birth. *Nature*, pages 1–10, 2024.
[21] Wei-Lin Qiu, Yu-Wei Zhang, Ye Feng, Lin-Chen Li, Liu Yang, and Cheng-Ran Xu. Deciphering pan­creatic islet *β* cell and *α* cell maturation pathways and characteristic features at the single-cell level. *Cell metabolism*, 25(5):1194–1205, 2017.
[22] X. Qiu, Q. Mao, Y. Tang, L. Wang, R. Chawla, H. A. Pliner, and C. Trapnell. Reversed graph embedding resolves complex single-cell trajectories. *Nature Methods*, 2017.
[23] Manu Setty, Vaidotas Kiseliovas, Jacob Levine, Adam Gayoso, Linas Mazutis, and Dana Pe’Er. Charac­terization of cell fate probabilities in single-cell data with Palantir. *Nature biotechnology*, 37(4):45 1–460, 2019.
[24] Han L Tan, Shu-Ling Chiu, Qianwen Zhu, and Richard L Huganir. Grip 1 regulates synaptic plasticity and learning and memory. *Proceedings of the National Academy of Sciences*, 117(40):25085–25091, 2020.
[25] Barbara Treutlein, Qian Yi Lee, J Gray Camp, Moritz Mall, Winston Koh, Seyed Ali Mohammad Shariati, Sopheak Sim, Norma F Neff, Jan M Skotheim, Marius Wernig, et al. Dissecting direct repro­gramming from fibroblast to neuron using single-cell RNA-seq. *Nature*, 534(7607):391–395, 2016.
[26] Valter Tucci, Anthony R Isles, Gavin Kelsey, Anne C Ferguson-Smith, Marisa S Bartolomei, Nissim Benvenisty, De´borah Bourc’his, Marika Charalambous, Catherine Dulac, Robert Feil, et al. Genomic imprinting and physiological processes in mammals. *Cell*, 176(5):952–965, 2019.
[27] Antonio Vitobello, Benoit Mazel, Vera G Lelianova, Alice Zangrandi, Evelina Petitto, Jason Suckling, Vincenzo Salpietro, Robert Meyer, Miriam Elbracht, Ingo Kurth, et al. Adgrl1 haploinsufficiency causes a variable spectrum of neurodevelopmental disorders in humans and alters synaptic activity and behavior in a mouse model. *The American Journal of Human Genetics*, 109(8):1436–1457, 2022.
[28] W. A. Whyte, D. A. Orlando, D. Hnisz, B. J. Abraham, C. Y. Lin, M. H. Kagey, P. B. Rahl, T. I. Lee, and R. A. Young. Master transcription factors and mediator establish super-enhancers at key cell identity genes. *Cell*, 153(2):307–319, 2013.

**Second round of review**

**Reviewer 1**

The authors have addressed my comments sufficiently.

**Reviewer 2**

Thank you for your efforts in revising the paper. some of my comments have been addressed. However, I still have several major concerns regarding the experimental data and its scalability compared to real-world data:
1. In Figure 10E, it appears that Sceptic_noCV (brown bar) outperforms the other methods by achieving the highest Kendall Tau value. However, the manuscript claims that Sceptic with cross validation (Sceptic_CV) is the best tool. Could the authors clarify this discrepancy?
2. In Figure 5, Sceptic seems to achieve a perfect accuracy (close to 1) across all simulation scenarios. I suspect this may be due to an oversimplification of the simulation procedure, perhaps by relying on only two arbitrary summary statistics.
3. It is weird that Sceptic’s accuracy remains consistently at 1 even when irrelevant genes are included. I suspect that the authors may have assigned similar expression profiles for these genes across time points, leading to their exclusion as highly variable genes. Further explanation on this matter would be required.
4. My major concern is the scalability of Sceptic for time-series single-cell data. The current strategy requires subsampling the number of cells at all time points to match the smallest time point. This strategy can be problematic when cell numbers vary significantly—for example, in the mouse gastrulation atlas, where cell counts range from 276 to 14,751. Subsampling all time points to 276 would result in a substantial loss of valuable data from time points with many more cells. Although the authors demonstrate that Sceptic outperforms other tools when using 200 cells per time point on the mouse embryogenesis atlas, I am not convinced that users would prefer Sceptic over the other tools if it necessitates discarding so much cellular information. I would also like to see a performance comparison of Sceptic with other tools on more complex datasets, such as the atlas of mouse embryogenesis, rather than solely presenting results for Sceptic.

**Authors Response**

**Point-by-point responses to the reviewers’ comments:**

**Reviewer #1**

The authors have addressed my comments sufficiently.

**Reviewer #2**

Thank you for your efforts in revising the paper. some of my comments have been addressed. However, I still have several major concerns regarding the experimental data and its scalability compared to real-world data:

1. In Figure 10E, it appears that Sceptic noCV (brown bar) outperforms the other methods by achieving the highest Kendall Tau value. However, the manuscript claims that Sceptic with cross validation (Sceptic CV) is the best tool. Could the authors clarify this discrepancy?

We believe that the results reported for Sceptic noCV reflect overfitting and hence do not reflect the true performance of the model. Our method, Sceptic CV, incorporates nested cross-validation to prevent overfit-ting, thereby ensuring more reliable performance on unseen data. The only reason we included Sceptic noCV in the empirical comparison is to enable a direct comparison to psupertime, which also leaves out the cross-validation step. The comparison between Sceptic CV and Sceptic noCV further illustrates how overfitting can inflate performance metrics. We have clarified the corresponding text as follows:

In this analysis, we included two variants of Sceptic, one that includes the cross-validation procedure (Scep­tic CV) and one without cross-validation (Sceptic noCV). The latter is included only to enable a direct comparison with Psupertime, which also leaves off the cross-validation step. We observe that Sceptic with­out cross-validation (Sceptic noCV) achieves a higher Kendall’s τ coefficient than Psupertime (Figure 3E). The fact that Sceptic noCV outperforms Sceptic CV is attributable to the fact that the former is effectively being evaluated on the same data that was used to train the model.

2. In Figure 5, Sceptic seems to achieve a perfect accuracy (close to 1) across all simulation scenarios. I suspect this may be due to an oversimplification of the simulation procedure, perhaps by relying on only two arbitrary summary statistics.

We agree with the reviewer’s concern that Sceptic performs exceptionally well in the simulations while psu-pertime does not. While we faithfully reproduced psupertime’s simulation framework, we acknowledge that the choice of summary statistics could potentially influence performance outcomes for both methods. We have addressed this concern by systematically investigating how noise magnitude affects Sceptic’s perfor­mance, demonstrating that both accuracy and Kendall τ decrease predictably as noise increases. Further details about these experiments are provided in our response to point 3, below. These additional simulations, together with our extensive validation of Sceptic’s superior performance across multiple real-world datasets (scRNA-seq data, single-nucleus imaging data), provide strong evidence for Sceptic’s robustness.

3. It is weird that Sceptic’s accuracy remains consistently at 1 even when irrelevant genes are included. I suspect that the authors may have assigned similar expression profiles for these genes across time points, leading to their exclusion as highly variable genes. Further explanation on this matter would be required.

We thank the reviewer for this important observation. In our simulation experiments, we intentionally included all genes as input without pre-selecting highly variable genes. The irrelevant genes were simulated using random Gaussian distributions (N(0,1)) with no temporal pattern, making them distinguishable from

2

Figure 1: **Sceptic’s performance under various noise magnitudes in irrelevant genes.** To evaluate Sceptic’s robustness to irrelevant gene signals, we conducted additional experiments with varying noise magnitudes. We simulated irrelevant genes using Gaussian distributions with zero mean and increasing standard variance (σ^2^ ∈ {1, 4, 25}). For each noise level, we generated 10 different datasets with random seeds and measured both accuracy and Kendall’s τ. As expected, Sceptic’s performance gradually decreases with increasing noise magnitude (Figure 1). These findings highlight both Sceptic’s robustness and its limitations under different noise scenarios.

temporally informative genes. In our initial experiments, we used standard Gaussian noise (σ^2^=1), which Sceptic could effectively identify and ignore, explaining the consistently high accuracy. To address the reviewer’s concern, we have now systematically tested Sceptic’s robustness by increasing the noise magnitude (σ^2^ ∈ {1, 4, 25}). As shown in Figure 1 (Supplementary Figure 9), both Sceptic’s accuracy and Kendall τ decrease predictably as noise magnitude increases, with accuracy dropping. These results (averaged across 10 random seeds) demonstrate that Sceptic’s performance is indeed sensitive to noise levels while maintaining robust performance under reasonable noise conditions.

4. My major concern is the scalability of Sceptic for time-series single-cell data. The current strategy requires subsampling the number of cells at all time points to match the smallest time point. This strategy can be problematic when cell numbers vary significantly—for example, in the mouse gastrulation atlas, where cell counts range from 276 to 14,751. Subsampling all time points to 276 would result in a substantial loss of valuable data from time points with many more cells. Although the authors demonstrate that Sceptic outperforms other tools when using 200 cells per time point on the mouse embryogenesis atlas, I am not convinced that users would prefer Sceptic over the other tools if it necessitates discarding so much cellular information. I would also like to see a performance comparison of Sceptic with other tools on more complex datasets, such as the atlas of mouse embryogenesis, rather than solely presenting results for Sceptic.

We agree that scaling Sceptic to larger datasets is an important and worthwhile goal. To this end, we imple­mented two key improvements. First, we implemented an option in Sceptic to employ XGBoost with GPU acceleration in place of the SVM. Second, inspired by Monocle’s use of low-dimensional embeddings (e.g., UMAP), we performed our tests using the top 30 principal components (PCs). This dimensionality reduction improved computational efficiency while preserving key biological variation. The improved Sceptic can now handle larger datasets, for example, completing analysis of the TOME dataset in under 30 minutes. This enables a more comprehensive assessment of Sceptic’s robustness and applicability to complex time-series

3

single-cell data. We conducted benchmarking against several established methods (psupertime, Monocle3, Palantir, and DPT) on the same dataset. However, we encountered significant computational challenges: DPT failed to complete after running for 10 days, Monocle3 was terminated most likely due to exceeding memory limits (500GB), and only Palantir successfully completed the analysis. Despite finishing, Palantir achieved a substantially lower Kendall τ correlation (-0.01) compared to Sceptic’s robust performance (0.74), demonstrating our method’s superior effectiveness and computational efficiency. These changes are described as follows:

**Scaling up Sceptic to large datasets**

To enhance computational efficiency and scalability, we implemented an option in Sceptic to use XGBoost [1] with GPU acceleration as an alternative to the default SVM classifier. The XGBoost implementation takes the same cell-by-measurement matrix as input and outputs cell-specific class probabilities. These probabilities are then used to compute pseudotime values via the same conditional expectation framework applied in the original SVM-based method.

To demonstrate the scalability of the enhanced Sceptic model, we performed pseudotime analysis on the mouse embryonic development dataset [2]. This dataset comprises single-nucleus transcriptomes from 12.4 million cells across 83 precisely staged mouse embryos, capturing the full course of prenatal development from late gastrulation (E8) to birth at high temporal resolution. To balance the number of cells across different time points, we downsampled the oversampled stages to match the time point with the fewest cells. Specifically, we randomly selected 46,253 cells from each of the 42 embryonic stages (excluding P0) and used the resulting balanced dataset (1.9 million cells in total) for pseudotime inference. We found that Sceptic accurately positions most cells near the diagonal in the pseudotime versus observed time plot, reflecting the temporal proximity of neighboring stages (Figure 2A). This result highlights Sceptic’s ability to preserve fine-grained temporal structure, even when the true time labels are closely spaced. We also generated a UMAP embedding colored by Sceptic-inferred pseudotime, which clearly illustrates the continuous temporal progression of cells along the trajectory (Figure 2B–C). Together, these results demonstrate Sceptic’s ability to scale to large, multi-cell-type datasets and its potential to generalize across complex time-series single-cell data.

**References**

[1] T. Chen and C. Guestrin. XGBoost: A scalable tree boosting system. In *Proceedings of the 22nd ACM SIGKDD International Conference on Knowledge Discovery and Data Mining*, KDD ’16, pages 785–794, New York, NY, USA, 2016. ACM.
[2] Chengxiang Qiu, Beth K Martin, Ian C Welsh, Riza M Daza, Truc-Mai Le, Xingfan Huang, Eva K Nichols, Megan L Taylor, Olivia Fulton, Diana R O’Day, et al. A single-cell time-lapse of mouse prenatal development from gastrula to birth. *Nature*, pages 1–10, 2024.

Figure 2: **Sceptic applied to an atlas of mouse embryogenesis. A** Confusion matrix for Sceptic on the mouse embryogenesis atlas data. The color scale is the row-normalized cell counts. FIXME: Change y-axis to “Embryonic day” and x-axis to “Sceptic pseudotime.” Clarify in the label what the color scale is. Remove the numeric values from the heatmap and make the whole heatmap smaller. Consider changing the maximum color scale to the 95th percentile of the values in the matrix, to improve readability. If you do this, say so in the caption. —Bill Fixed. **B** UMAP of mouse embryogenesis cells, colored by cell type. **C** UMAP of mouse embryogenesis cells, colored by Sceptic’s inferred pseudotime.

**Third round of review**

**Reviewer 2**

The authors have addressed my concerns and I do not have any further comments.
